# Micro-scale screening of genetically modified *Fusarium fujikuroi* strain extends the apicidin family

**DOI:** 10.1007/s13659-024-00473-9

**Published:** 2024-08-23

**Authors:** Alica Fischle, Mika Lutsch, Florian Hübner, Linda Schäker-Hübner, Lina Schürmann, Finn K. Hansen, Svetlana A. Kalinina

**Affiliations:** 1 Institute of Food Chemistry, University of Münster, Corrensstraße 45, 48149 Münster, Germany; 2 Graduate School of Natural Products, Corrensstraße 43, 48149 Münster, Germany; 3 Pharmaceutical Institute, Pharmaceutical and Cell Biological Chemistry, University of Bonn, An Der Immenburg 4, 53121 Bonn, Germany

**Keywords:** Cyclic tetrapeptides, Apicidins, Cytotoxicity, HDAC inhibition, Tropical diseases

## Abstract

Apicidins are a class of naturally occurring cyclic tetrapeptides produced by few strains within the *Fusarium* genus. These secondary metabolites have gained significant attention due to their antiprotozoal activity through HDAC inhibition, thereby highlighting their potential for the treatment of malaria. Predominantly, apicidins have been isolated from *Fusarium semitectum*, offering a deep insight into the biosynthetic pathway responsible for their formation. A similar biosynthetic gene cluster has also been identified in the rice pathogenic fungus *F. fujikuroi*, leading the discovery of three additional apicidins through genetic manipulation. Routine mass spectrometric screening of these compound-producing strains revealed another metabolite structurally related to previously studied apicidins. By optimizing culture conditions and developing an effective isolation method, we obtained a highly pure substance, whose chemical structure was fully elucidated using NMR and HRMS fragmentation. Further studies were conducted to determine cytotoxicity, antimalarial activity, and HDAC inhibitory activity of this new secondary metabolite alongside the previously known apicidins. This work not only expands the apicidin class with a new member but also provides extensive insights and comparative analysis of apicidin-like substances produced by *F. fujikuroi*.

**Graphical Abstract:**

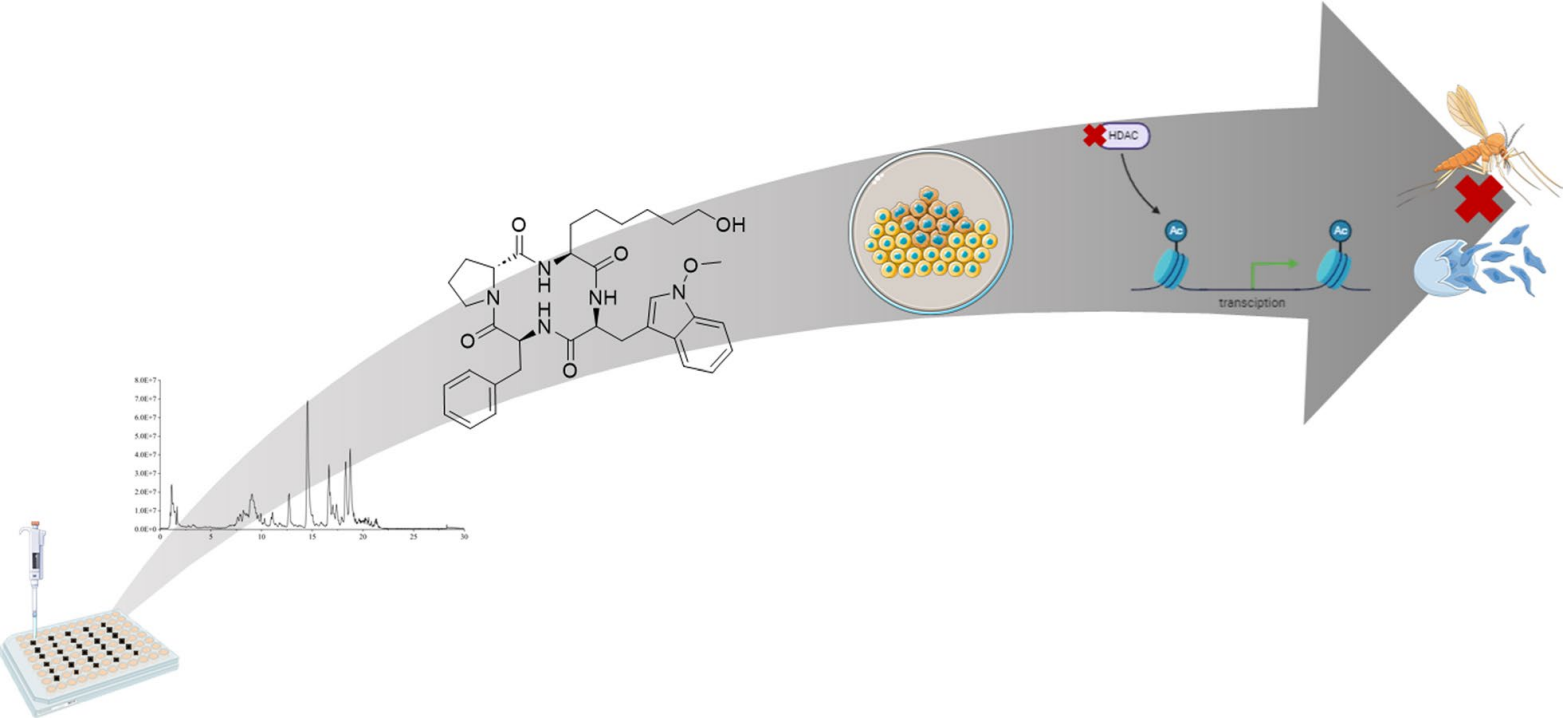

**Supplementary Information:**

The online version contains supplementary material available at 10.1007/s13659-024-00473-9.

## Introduction

**Fig. 1:**
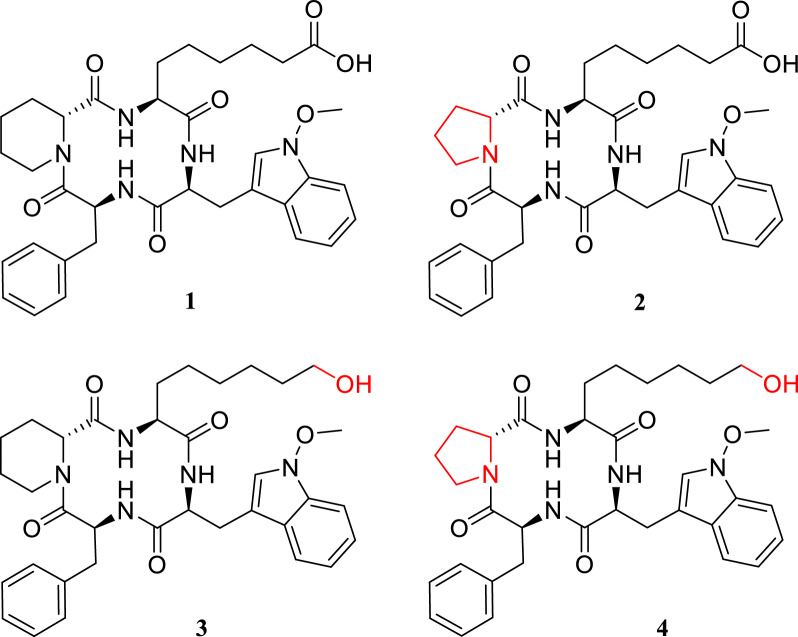
Chemical structures of known apicidins from *Fusarium fujikuroi*: apicidin F (**1**), apicidin J (**2**), apicidin K (**3**), and the new metabolite apicidin L (**4**). Structural differences are highlighted in red

In recent years, cyclic peptides have aroused keen interest to develop new treatment options for a broad range of diseases [1–3], among them also cyclic tetrapeptides (CTP) [4–6]. Their unique structure in certain biological systems enhances properties like biostability and bioavailability, leading to improved overall bioactivity [7, 8]. This structural versatility also offers numerous opportunities for chemical modifications through the incorporation and alteration of various functional groups within the peptide framework [4]. However, these modifications have proven challenging to implement, and total synthesis of new CTPs has faced substantial difficulties [7–10]. Despite these challenges, cyclic peptides have been extensively researched and obtained from various organisms including plants [11, 12], insects [11], and fungi [13], utilizing the organisms’ natural biosynthetic pathways for novel compounds discovery and their diverse biological activities. Among fungal CTPs, the class of apicidins has drawn increased attention due to their antiprotozoal activity through inhibition of the parasitic HDACs [14], which is of high relevance in the discovery of new active compounds for treatment of diseases like malaria tropica. Following this discovery, genetic modifications of *Fusarium semitectum* and *F. sambucinum* have led to the expansion of the apicidin class, as well as to new insights into the biosynthetic gene cluster (BGC) responsible for the production of the apicidins, including allocation of the respective gene functions [15–17]. Additionally, the BGCs correlated to the production of apicidins have been identified in *F. incarnatum* and *F. scirpi* [18], and investigations of accessory chromosomes in *F. poae* also allowed detection of various apicidins [19]. Comparable investigations showed a similar yet silent BGC in the rice pathogen *F. fujikuroi*, and genetic investigations coupled with chemical analysis allowed not only the identification of the BGC [20] and its specific functions yielding a proposed biosynthetic pathway [21] but more importantly the discovery of a new member of the apicidin class: apicidin F (**1**), which was identified as 6-[(3*S*,6*S*,9*S*,12*R*)-3-benzyl-6-[(1-methoxyindol-3-yl)methyl]-2,5,8,11-tetraoxo-1,4,7,10-tetrazabicyclo[10.4.0]hexadecane-9-yl]hexanoic acid (see Fig. 1). This was confirmed via NMR as well as various mass spectrometric techniques [20–22]. It was shown that the BGC consists of twelve genes, where *APF1* was confirmed to transcribe for the non-ribosomal peptide synthase (NRPS) as core enzyme guiding the proposed biosynthetic pathway, as this enzyme was shown to be responsible for the incorporation and cyclization of the four amino acids that constitute apicidin F (**1**): d-pipecolic acid, l-2-aminooctanedioic acid, *N*-methoxy-l-tryptophane, and l-phenylalanine. Furthermore, the function of *APF3* as Δ^1^-pyrroline-5-carboxylate reductase was associated with the modification of l-lysine to d-pipecolic acid, and *APF9*’s function as FAD-dependent monooxygenase seems to be involved in the transformation of the hydroxy-group to the carboxylic acid of l-2-aminooctanedioic acid [21]. Apicidin F (**1**) was further tested for cytotoxicity on a human liver carcinoma cell line (HepG2) and investigated for its antimalarial activity [21, 22]. In HepG2, the IC_50_ was determined at 110 µM [21], and the compounds’ antiparasitic activity against *Plasmodium falciparum* was observed at an IC_50_ of 0.67 µM [22]. In contrast, apicidin itself presents an IC_50_ of 1.3 µM in HepG2 [21] and 0.2 µM in the *P. falciparum* assay [14, 22].

Alongside apicidin F (**1**), two more compounds were identified in the genetically modified strains, which were assigned as apicidin J (**2**) (6-[(3*S*,6*S*,9*S*,12*R*)-3-benzyl-6-[(1-methoxyindol-3-yl)methyl]-2,5,8,11-tetraoxo-1,4,7,10-tetrazabicyclo[10.3.0]pentadecane-9-yl]hexanoate) and apicidin K (**3**) ((3*S*,6*S*,9*S*,12*R*)-3-benzyl-9-(6-hydroxyhexyl)-6-[(1-methoxyindol-3-yl)methyl]-1,4,7,10-tetrazabicyclo[10.4.0]hexadecane-2,5,8,11-tetrone) [21]. The structures of both compounds (see Fig. 1) were elucidated successfully and are shown to differ from apicidin F (**1**) only by implementation of D-proline instead of D-pipecolic acid (apicidin J, **2**) or by 2-amino-8-hydroxyoctanoic acid instead of L-2-aminooctanedioic acid (apicidin K, **3**) [21]. However, the yield of both compounds was not sufficient to carry out investigations regarding cytotoxicity and potential bioactivity. Additionally, while the structure for apicidin J (**2**) was confirmed with MS-techniques, it was not possible to obtain a full NMR-spectrum [21]. Therefore, in this study, two different mutant strains of *F. fujikuroi* were cultured for the initial investigations, an overexpression (OE) of the bANK transcription factor (OE::*APF2*) and a deletion (Δ) and OE-mutant Δ*APF9/*OE::*APF2* alongside the wild-type (WT) strain. The initial investigations were conducted to enhance the fungus’ production through optimization of the cultivation conditions for the three known apicidins F (**1**), J (**2**), and K (**3**), while simultaneously screening for an apicidin-like structure carrying both proline and 2-amino-8-hydroxyoctanoic acid modifications (see Fig. 1). The proposed compound to be fully characterized is initially referenced as apicidin L (**4**). This approach was chosen to allow for completion of the structure elucidation of apicidin J (**2**) via NMR. Moreover, an additional emphasis was placed on the cytotoxicity on various cell lines and biological activity towards antiparasitic properties through the inhibition of HDACs of all four apicidin-derivatives. Overall, this study provides prove of the structural configuration of apicidin J (**2**) and K (**3**) via NMR, and a full analytical structure elucidation of novel apicidin L (**4**). Furthermore, the cytotoxicity of all compounds (**1–4**) is provided for HepG2 and A549 (human lung adenocarcinoma). The biological activity of the isolated substances toward *P. falciparum* shows apicidin F (**1**) to be the most active compound, and investigations of HDAC inhibitors revealed the most potent IC_50_ for apicidin F (**1**) and apicidin J (**2**) against HDAC1 and HDAC2.

## Results and discussion

### Semi-qualitative micro-scale screening of apicidin-like metabolites from *Fusarium fujikuroi*

**Fig. 2:**
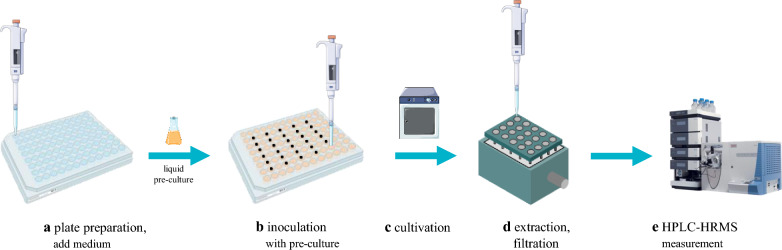
Schematic procedure of micro-scale cultivation in 96-well plates. First, the plate is prepared (**a**) by adding 150 µL of agarose-containing medium to each well. Upon solidification of the media, inoculation (**b**) occurs with 50 µL of liquid pre-culture to every second column and leaving the outermost colums empty. After suitable incubation (**c**) the inoculated colums are extracted (**d**) twice with 80/20 MeOH/H_2_O (v/v) and filtered through a 96-well plate filter under reduced pressure utilizing a vacuum manifold and collected in a fresh 96-well plate, which was then directly transferred for HPLC-HRMS (**e**) measurement. The figure was prepared with biorender.com and smart.servier.com

The new metabolite was identified during the routine screening of the available genetically modified strains from *F. fujikuroi* [20, 21], Δ*APF1*, OE::*APF2,* Δ*APF3*/OE::*APF2*, and Δ*APF9/*OE::*APF2* together with the wild-type strain. Here, an established micro-scale cultivation in 96-well plates was utilized. This method allows less material and chemical consumption, shorter cultivation time, and more efficient sample preparation, thereby keeping it inexpensive. Additionally, up to five experiments can be performed simultaneously while maintaining a high number of biological replicates (*N* = 6 per column). This procedure follows five basic steps, which are given in Fig. 2. In order to keep fungal interaction through volatile substances to a minimum and gain insight into each strains’ specific secondary metabolites, each 96-well plate is inoculated with solely one strain.

**Fig. 3:**
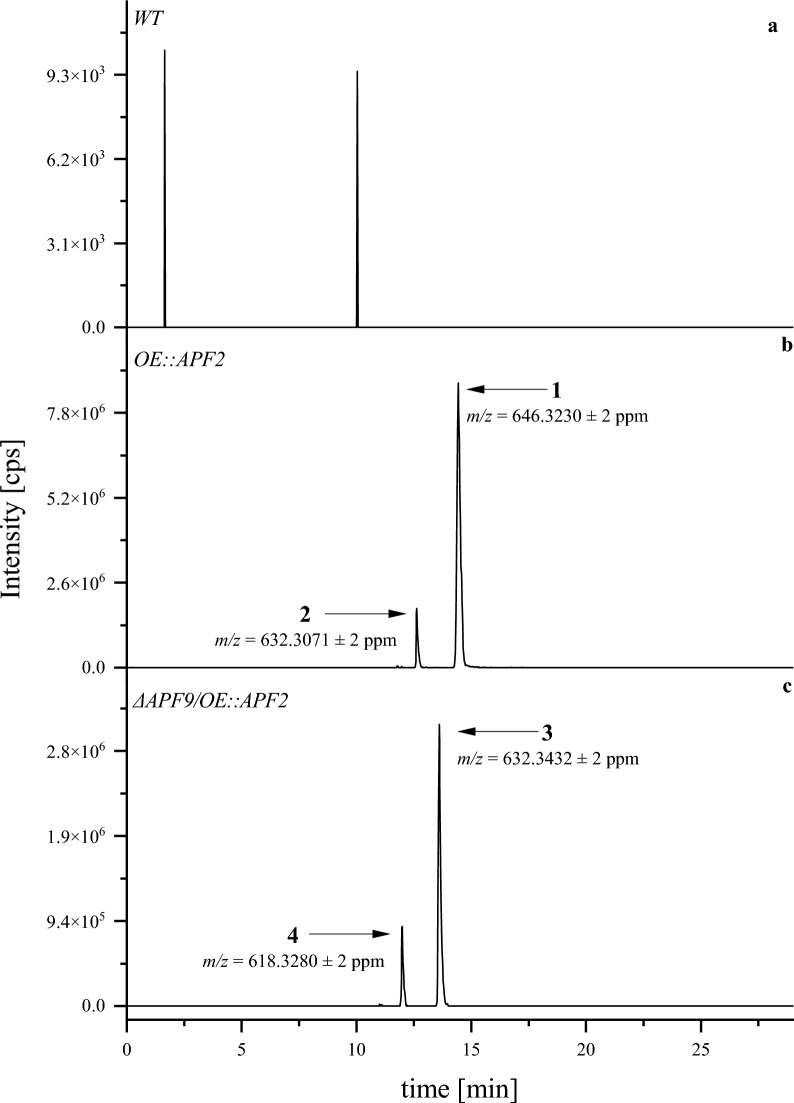
Extracted ion chromatograms (XIC) for the known apicidins F (**1**), J (**2**), K (**3**), and the newly detected apicidin L (**4**) in wild-type (**a**) and mutant strains (**b,c**) after cultivation in micro-scale on DVK for 4 days at 28 °C at saturated humidity in the dark. The XICs were generated based on the exact mass-to-charge-ratio (*m/z*) ± 2 ppm as given in the figure

As shown in Fig. 3, HRMS detection after liquid chromatographic separation allowed the assignment of the three known apicidins F (**1**), J (**2**), and K (**3**). Additionally, the data was screened for a potential new metabolite carrying both modifications of proline and 2-amino-8-hydroxyoctanoic acid. As expected, apicidin F (**1**) and J (**2**) were identified in the mutant OE::*APF2* which transcribes for the specific bANK-transcription factor [21]. Furthermore, apicidin K (**3**) was identified in the deletion- and OE-mutant Δ*APF9/*OE::*APF2* which carries two genetic modifications; the deletion of the FAD-dependent monooxygenase *APF9*, and the identical overexpression of the transcription factor *apf2* [21]. Here, the new metabolite, apicidin L (**4**), was first identified.

One way to enhance production of fungal secondary metabolites and thereby increase formation of the new apicidin L (**4**) is by modification of the growth medium [23–25]. Therefore, miniature-scale supplementation of single and combined amino acids was conducted as a micro-scale screening in 96-well plates. The amino acids were chosen based on the proposed biosynthetic pathway of apicidin F (**1**) [21], resulting in a total of five supplements: l-tryptophane (l-trp), l-phenylalanine (l-phe), l-lysine (l-lys), l-glutamate (l-glu) as proline precursor, and d/l-2-aminooctanoic acid (2-aoa). Each amino acid was supplemented directly to the autoclaved medium to yield a final concentration of 50 mM. Additionally, combined amino acid compositions were tested. The extracts were filtered and directly used for HPLC-HRMS measurement.

**Fig. 4:**
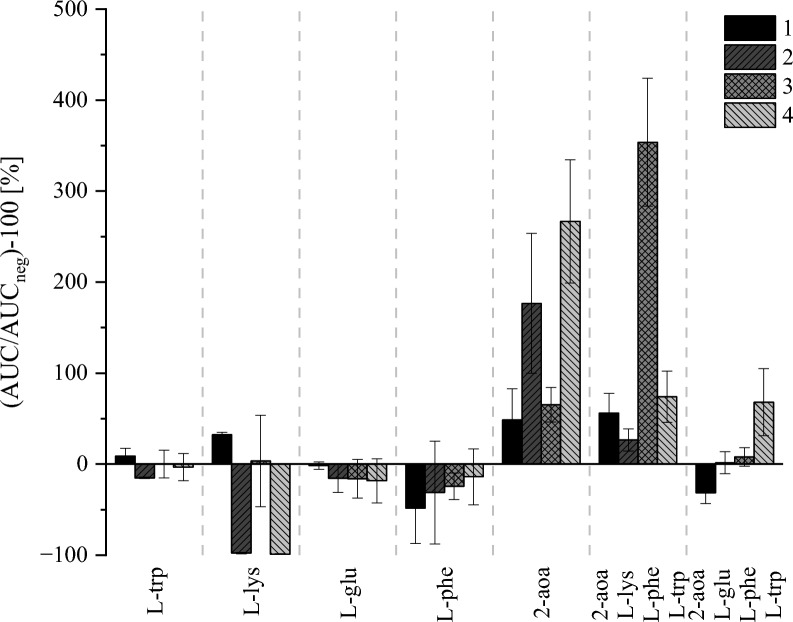
Relative formation of the four apicidins in medium supplemented with various amino acids (50 mM) in relation to non-supplemented medium. The Y-axis depicts the obtained area under the curves (AUC) after peak integration placed in relation to negative control (AUC_neg_) without supplementation in %. Black columns show apicidin F (**1**), dark grey columns show apicidin J (**2**), grey columns show apicidin K (**3**), and light grey column show the new apicidin L (**4**). Each experiment has *N* = 6 replicates cultivated for 5 d at 28 °C in the dark at saturated humidity, given are mean values ± standard deviations in %

The results in Fig. 4 show that for the single amino acid supplementation, d/l-2-aoa had the most pronounced impact on the production of apicidin J (**2**) and apicidin L (**4**), with apicidin-F (**1**) and apicidin K (**3**) also being produced in higher amounts compared to the production in non-supplemented medium. Interestingly, only one of the combined amino acid supplementations enhanced fungal secondary metabolite production toward overall compound formation, with d/l-2-aoa, l-lys, l-phe, and l-trp boosting especially production of apicidin K (**3**). The reasoning here needs to consider the unfavorable conditions caused by providing l-lys to the medium, as lysine serves as a precursor for the incorporation of pipecolic acid [21]. In this case, the biosynthesis might be shifted in favor of apicidin F (**1**) and apicidin K (**3**), while suppressing the formation of apicidin J (**2**) and the new apicidin-like structure (**4**). Combining this effect with an inducing effect by d/l-2-aoa and considering that the used strain is missing a functional monooxygenase [21], the second oxidation step required to convert l-2-aminooctaneoic acid to l-2-aminooctanedioic acid cannot be executed, thereby, direct incorporation of the intermediate product might be pushed the biosynthesis towards apicidin K (**3**). Therefore, it is reasonable that the single supplementation of d/l-2-aoa increased the production of the new metabolite, considering that the monooxygenase activity is absent. Consequently, another experiment was conducted to determine the optimal d/l-2-aoa concentration of the supplement by preparing standard agar plates with 10, 20, 30, 40, and 50 mM. After 5 days of cultivation, a representative square of 1 cm^2^ of mycelium was extracted with 80/20 MeOH/H_2_O (v/v) and analyzed by HPLC-HRMS.

**Fig. 5:**
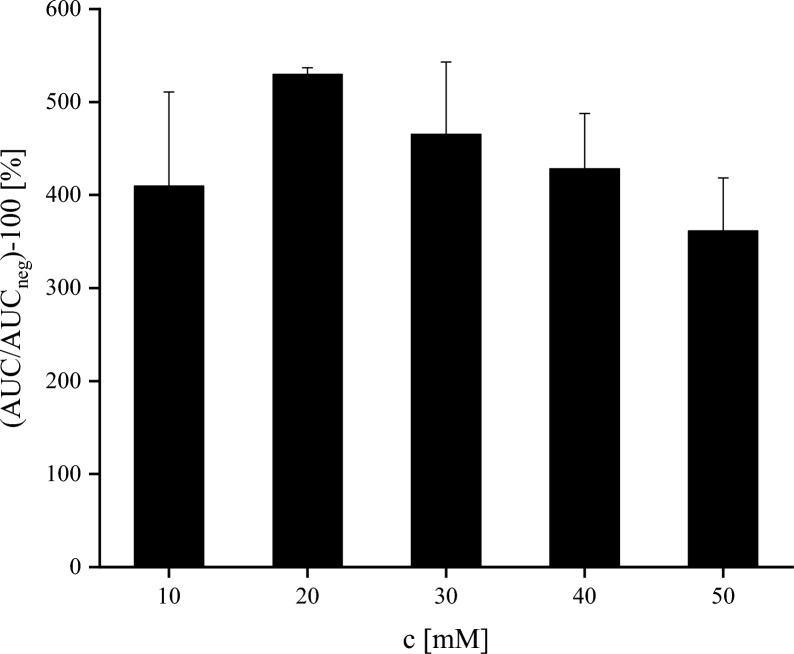
Determination of optimal supplementation concentration range for the new apicidin L (**4**). The Y-axis depicts the obtained AUCs after peak integration shown in relation to the negative control without supplementation (AUC_neg_). The X-axis shows the concentration of 2-aoa supplemented to the medium in mM. Experiments were conducted with *N* = 3 replicates. The columns depicted are mean values of AUC ± standard deviation in %

**Fig. 6:**
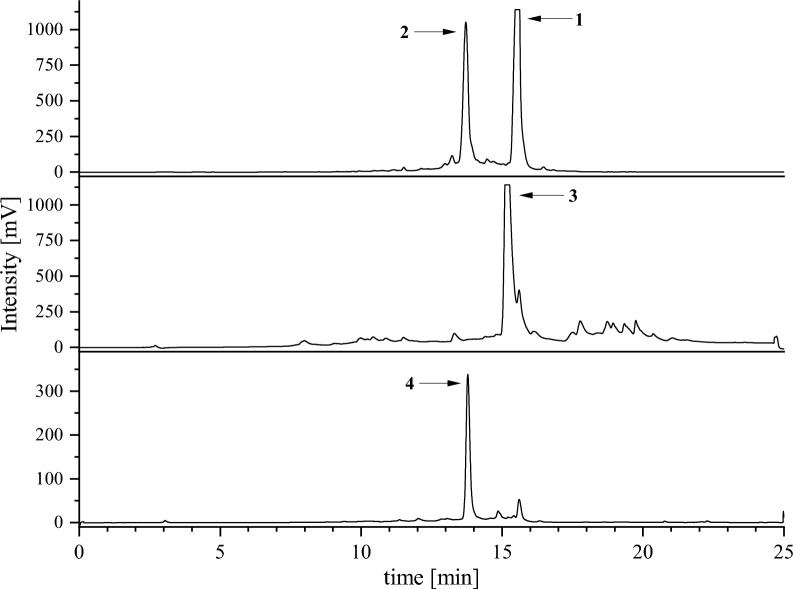
Semi-preparative HPLC–UV-chromatograms after SPE processing. Apicidin F (**1**) eluting at 15.4 min and apicidin J (**2**) eluding at 13.7 min were purified from combined SPE fractions, while apicidin K (**3**) eluting at 15.2 min and apicidin L (**4**) eluting at 13.6 min were separated successfully on the SPE and processed individually. Chromatographic separation was achieved on a ReprosilPur 120 C_18_-AQ column (250 × 10 mm, Dr. Maisch GmbH) equipped with a C_18_ guard column (4 × 3 mm, Phenomenex) and recorded at λ = 225 nm

Based on the AUC shown in Fig. 5, a concentration of 20 mM supplement was determined as suitable and applied to laboratory-scale cultivation of the fungal strains. After five days of cultivation time, large-scale extraction was performed with ethyl acetate followed by SPE purification using a MeCN/H_2_O gradient. Apicidin F (**1**) and apicidin K (**3**) were present in the late 50/50 MeCN/H_2_O (v/v) and the 100% MeCN fractions while apicidin J (**2**) and the new metabolite (**4**) eluted only early within 50/50 MeCN/H_2_O (v/v). Exemplary chromatograms of the semi-preparative HPLC–UV applied are depicted in Fig. 6.

Each of the isolated compounds had a purity ≥ 95% (HPLC-ELSD). The respective yields were 7.5 mg for apicidin F (**1**), 7.9 mg for apicidin J (**2**), 13.3 mg for apicidin K (**3**), and 25.2 mg for apicidin L (**4**).

### Structural investigations

**Fig. 7:**
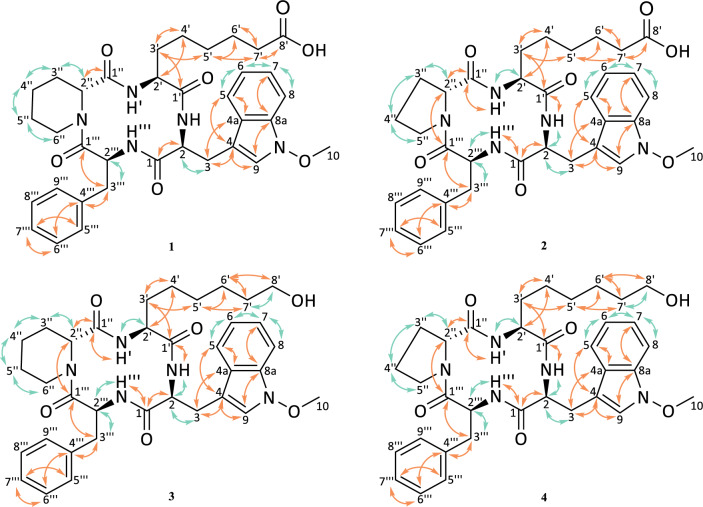
Chemical structures of the purified secondary metabolites apicidin F (**1**, in CD_3_OD), apicidin J (**2**, in C_5_D_5_N), apicidin K (**3**, in C_5_D_5_N), and apicidin L (**4**, in C_5_D_5_N) based on 1D- and 2D-NMR experiments and comparison to previous studies [21, 22]. Orange arrows indicate HMBC correlations, green arrows show COESY correlations. Atom numbering according to IUPAC

For NMR analysis, C_5_D_5_N was used as solvent for apicidin J (**2**), K (**3**), and L (**4**). In contrast, apicidin F (**1**) was dissolved in CD_3_OD. The recorded experiments included ^1^H-NMR, ^13^C-NMR, COSY, HMBC, HSQC, and ROESY. The evaluated COSY and HMBC correlations are presented in Fig. 7. Structures of apicidin F (**1**) and apicidin K (**3**) were in agreement with previously published investigations [21, 22]. Since NMR analysis for apicidin J (**2**) remained open [21], its chemical structure was also confirmed. For the new metabolite (**4**), a chemical formula of C_34_H_43_N_5_O_6_ was proposed from HRMS screenings which were allocated to tryptophane, proline, phenylalanine, and 2-amino-8-hydroxyoctanoic acid. Typically, the amide carbons were found between δ_C_ = 172.0 ppm and 177.0 ppm. In correspondence, CHα in combination with NH doublets were observed around δ_H_ = 7.4–9.6 ppm and assigned by COSY investigations (Table 1). The aromatic signals observed at δ_C_ = 130.0 ppm and δ_H_ = 7.4 ppm confirmed the presence of the phenylalanine-moiety. Furthermore, HMBC investigations allowed allocation of aromatic hydrogen- and carbon-atoms toward the tryptophane-moiety. Here, an additional singlet was observed at δ_C_ = 66.2 ppm and δ_H_ = 3.9 ppm, indicating the presence of a methoxy group at the tryptophane. Its binding to the nitrogen present in the indole-ring was confirmed by ROESY-experiments, where the spatially adjacent hydrogens were shown to correlate between H-9 and H-10. Additionally, no NH-signal was present. Next, the occurrence of 2-amino-8-hydroxyoctanoic acid was examined. Here, a comparison with apicidin K (**3**) was conducted [21]. Indeed, low field shifts at the 8′ position were observed at δ_C_ = 62.5 ppm and δ_H_ = 3.8 ppm, confirming the linkage to a hydroxy moiety present at δ_H_ = 5.1 ppm. Additionally, the presence of a six-carbon long sidechain was proven by HMBC correlations for high-field shifted CH_2_. Finally, the presence of proline remained to be investigated. Based on literature [26], starting from the last remaining CHα at 3″, two high-field and one low-field shifted CH_2_-groups were observed at δ_C_ = 25.3–25.4 ppm and δ_C_ = 47.2 ppm, respectively [26]. The linkage of the later CH_2_ to the last remaining nitrogen is indicated by δ_H_ = 4.1 ppm and 3.4 ppm, which confirmed the proline-moiety. Thereby, strong evidence is provided for the chemical structure initially proposed for the new apicidin-like compound (**4**). An overview of the COSY and HMBC-correlations is given in Fig. 7.

**Table 1:** NMR data for novel apicidin L (**4**)

C_5_D_5_N		
	Apicidin L	
Position	*δ*_C_/ppm, type	*δ*_H_/ppm, m, (J in Hz)
***N*-methoxytryptophane**		
NH		9.96, d (6.7)
1	175.1, C	
2	62.5, CH	4.48, dt (9.6, 7.0)
3	26.0, CH_2_	4.24–4.14, m 3.87, dd (14.5, 7.3)
4	108.7, C	
4a	124.7, C	
5	119.9, CH	7.74, d (7.9)
6	120.7, CH	7.18, t (7.0)
7	123.4, CH	7.36–7.30, m
8	109.4, CH	7.53, d (8.1)
8a	133.4, C	
9	123.3, CH	7.50–7.43, m
10	66.2, CH_3_	3.91, s
**2-amino-8-hydroxy-octanoic acid**		
NH′		7.86, d (10.0)
1′	176.7, C	
2′	55.8, CH	4.75, ddd (10.0, 8.4, 6.8)
3′	30.9, CH_2_	2.06–1.89, m 1.66–1.60, m
4′	26.7, CH_2_	1.30–1.18, m
5′	29.9, CH_2_	1.30–1.18, m
6′	26.6, CH_2_	1.50–1.31, m
7′	34.1, CH_2_	1.66–1.60, m
8′	62.4, CH_2_	3.81, t (6.5)
OH′		5.10, s
**proline**		
1″	172.4, C	
2″	58.6, CH	5.03–4.95, m
3″	25.5, CH_2_	2.38–2.30, m 1.50–1.38, m
4″	25.4, CH_2_	2.11, dt (18.0, 6.8) 1.60–1.51, m
5″	47.2, CH_2_	4.11, dd (9.6, 3.9) 3.35, dt (10.1,8.1)
**Phenylalanine**		
NH‴		8.55, d (10.1)
1‴	173.9, C	
2‴	54.3, CH	5.61, ddd (10.2, 8.7, 6.8)
3‴	37.2, CH_2_	3.54, dd (13.6, 8.7) 3.28, dd (13.6, 6.8)
4‴	138.6, C	
5/9‴	130.2, CH	7.50–7.43, m
6/8‴	129.4, CH	7.36–7.30, m
7‴	127.5, CH	7.25, t (8.1)

^1^H NMR was recorded at 600 MHz, ^13^C NMR was recorded at 150 MHz in C_5_D_5_N

Next, the amino acid sequence of the new metabolite was determined. Previous investigations on apicidin F (**1**) [21, 22], apicidin J (**2**) [21], and apicidin K (**3**) [21] were unable to prove the amino acid sequence via NMR, instead, hydrolysis and MS-experiments were utilized to confirm the sequence of the amino acids within the CTP [21]. Since NMR-measurements were re-conducted in C_5_D_5_N, HMBC correlations allowed to assign the amide protons to their respective carbonyl carbon, resulting in depiction of a 12-membered macrocyclic ring for all four compounds with identical amino acid sequence. Thereby, the amino acid sequence of the new metabolite was additionally verified by ROESY correlations detected between proline”Hα/ 2-amino-8-hydroxyoctanoic acid′NH, 2-amino-8-hydorxyoctanoic acid′Hα/*N*-methoxy-tryptophane-NH, *N*-methoxy-tryptophaneHβ/ phenylalanine‴NH, and phenylalanine‴Hα/ proline''Hδ. With the verified amino acid sequence, the core structure of the new metabolite was proven to consist of *N*-methoxy-tryptophane-2-amino-8-hydroxyoctaonic acid-proline-phenylalanine.

Furthermore, investigations regarding the amino acid configuration were conducted. Previous investigations on apicidin F (**1**) [21, 22], apicidin J (**2**) [21], and apicidin K (**3**) [21] determined the configuration of each amino acid in the peptide framework by Marfey’s derivatization and HPLC-HRMS measurement. Subsequent comparison of the retention times obtained from the hydrolysate to the chromatographically separated d- and l-configured reference material allowed the allocation of one d-configured amino acid (pipecolic acid or proline) while all other amino acids were l-configured. For apicidin L (**4**), the configuration was determined by comparison of the proton coupling constants to those observed for apicidin K (**3**) [21]. Similar to Suciati el al., the Hα of *N*-methoxy-l-tryptophane showed vicinal coupling to the adjacent NH with ^3^*J*_HαNH_ = 6.7 Hz, which indicated a *syn*-relationship [16]. Coupling of the Hα in 2-amino-8-hydoxyoctanoic acid (^3^*J*_Hα′NH′_ = 10.0 Hz) and phenylalanine (^3^*J*_Hα‴NH‴_ = 10.1 Hz) to the adjacent NH indicated an *anti*-relationship. Therefore, it is suggested that these three amino acids are l-configurated [16]. Additionally, a ROESY correlation between H-5″ and H-2″ was observed. Computational analysis of the distance between these two hydrogens via the software Chem3D ver. 22.2.0 revealed a distance of 2.7 Å for a d-configurated and 4.6 Å for a l-configurated proline. As typical ROESY couplings occur over a maximum distance of 5 Å and the intensities of cross peaks are expected to drop with farther spaced hydrogens, the presence of d-proline is strongly implied [27]. This is also supported by the proposed biosynthetic pathway of apicidin F (**1**). Here, the l-lysine serves as precursor for d-pipecolic acid [21], but it could also be incorporated to the CTP as d-proline [28], which is shown since none of the previously characterized apicidin F (**1**) [21, 22], apicidin J (**2**) [21], and apicidin K (**3**) [21] differed in the absolute configuration of d-pipecolic acid or d-proline. Niehaus et al. showed that the polyketide synthase *apf1* is responsible for the change in conformation from l- to d-pipecolic acid and subsequent assembly of the CTP [21], which is fully functional in the used mutant strains OE::*APF2* and Δ*APF9/*OE::*APF2*. In addition, the deletion of the FAD-dependent monooxygenase *apf9* does neither affect d-pipecolic acid nor d-proline, thus, no other configuration is suspected for apicidin L (**4**). Therefore, based on these data, the newly identified compound is determined to consist of four amino acids, named in order of their sequence and corresponding to their configuration, l-tryptophane-*N*-methoxy, l-2-amino-8-hydroxyoctanoic acid, d-proline, and l-phenylalanine. According to IUPAC, the nomenclature of this compound is suggested as (3*S*,6*S*,9*S*,14a*R*)-9-benzyl-3-(6-hydroxyhexyl)-6-[(1-methoxy-1H-indol-3-yl)methyl]decahydro-pyrrolo[1,2-a][1,4,7,10]tetraazacyclododecine-1,4,7,10-tetraone. It is assigned to the compound class of apicidins, and in accordance with the identified compounds of this class so far, its initially proposed trivial name is confirmed as apicidin L (**4**).

Furthermore, while the chemical structure of apicidin J (**2**) was investigated with MS-techniques, final confirmation with NMR remained open [21]. In order to complete this investigation, the measurement was re-attempted. The obtained data showed clear spectra with similarities to apicidin F (**1**) [22] and apicidin L (**4**). By comparison to apicidin F (**1**), the presence of l-2-aminooctanedioic acid was confirmed [22], while similarities to apicidin L (**4**) highly implied the incorporation of d-proline instead of d-pipecolic acid. This confirms the chemical structure of apicidin J (**2**) as suggested by Niehaus et al., and additionally supports their observations from the amino acid configuration through Marfey’s derivatization [21]. A comparison of COSY- and HMBC-correlations for each compound is presented in Fig. 7. The complete dataset for all measured and analyzed NMR spectra is given in the Additional file 1.

**Fig. 8:**
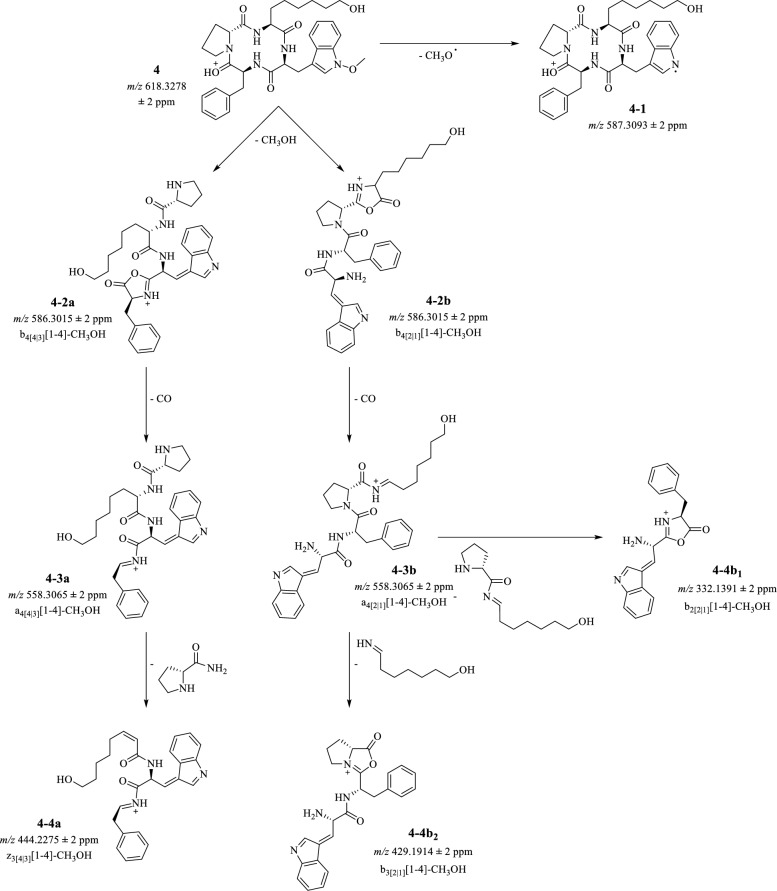
Proposed fragmentation pathway for apicidin L (**4**) in positive ionization mode, [M + H]^+^ 618.3278 ± Δm in ppm. Nomenclature follows the suggestions of Niedermeyer and Strohmann [30]. First, cleavage of the methoxy-group is observed (**4-1**), before or after opening of the ring between d-proline and l-phenylalanine or l-tryptophane and l-2-amino-8-hydroxyoctanoic acid (**4-2a**, **4-2b**). Next, loss of CO is observed from the oxazolone ring (**4-3a**, **4-3b**), and finally, the amino acid side chains are cleaved (**4-4a**, **4-4b**_**1**_, **4-4b**_**2**_)

As an additional tool to confirm apicidin L’s (**4**) chemical structure, MS^n^ fragmentation of the CTP was performed with a 15 µg/mL solution (80/20 MeCN/H_2_O (v/v) + 1% acetic acid). The compound was directly injected into the linear ion-trap coupled to a high-resolution mass spectrometer. The protonated mass was identified in positive ionization mode at *m/z* 618.3278 (**4**) and fragmented via CID (see Fig. 8). Since CTPs lack terminal N- and C-groups, the initial fragmentation is usually a ring-opening under formation of a C-terminal oxazolone ring [29]. For the new CTP, four fragmentation sites have to be considered. Initial fragmentation of *m/z* 618.3278 (**4**) resulted in two abundant signals at *m/z* 586.3015 (**4-2a, 4-2b**) and *m/z* 587.3093 (**4-1**), which can be assigned to the neutral loss of CH_4_O and an ∙OCH_3_ radical, respectively, indicating a cleavage of the methoxy-group at l-tryptophane, stabilizing the radical eventually through its aromatic ring system (see Fig. 8). Yet, only *m/z* 586.3015 (**4-2a, 4ab**) fragmented further. Here, the MS^3^ fragmentation showed the most abundant fragment at *m/z* 558.3065 (**4-3a, 4-3b**) which was assigned to a neutral loss of CO. Further MS^4^ fragmentation of this ion showed three fragments at *m/z* 429.1914 (**4-b**_**2**_), *m/z* 332.1391 (**4-b**_**1**_), and *m/z* 444.2275 (**4-4a**), sorted by the abundancy of their signals. Starting from the least abundant MS^4^-fragment of *m/z* 444.2275 (**4-4a**), the potential ring-opening locations could be determined as follows: based on this *m/z*, a cleavage of the d-proline-moiety together with a N-terminus is suggested. Considering the linkage between d-proline and l-2-amino-8-hydroxyoctanoic acid, an a → b fragmentation with a subsequent cleavage of NH_3_ would need to occur to yield a *m/z* 444.2275 (**4-4a**). However, since no NH_3_-containing intermediate was observed to support this hypothesis, the initial ring opening is presumably taking place at d-proline. Yet, the fragment *m/z* 429.1914 (**4-4b**_**2**_) suggests the neutral loss of l-2-amino-8-hydroxyoctanoic acid under an a → b fragmentation, thus indicating a ring-opening between this amino acid and l-tryptophane [29, 30]. Both sites are equally likely for the opening of the cyclic structure. Still, an opening between l-2-amino-8-hydroxyoctaonic acid and d-proline can be further disproven by the *m/z* 332.1391 (**4-4b**_**1**_) fragment, as this *m/z* corresponds to the cleavage of the linked amino acids. This fragment would not be observed if the ring-opening has taken place between these peptides. Additionally, the MS^4^ fragmentation of *m/z* 332.1391 (**4-4b**_**1**_) yields an *m/z* 304.1436 (Additional file 1) which aligns with a neutral loss of CO, proving *m/z* 332.1391 (**4-4b**_**1**_) as a b-ion. The suggested fragmentation pathway is summarized in Fig. 8.

As an additional means to investigate the absolute amino acid configuration, crystallization of apicidin L (**4**) was attempted. The results, however, yielded amorphous and non-defractable crystals. In case of apicidin itself, previous investigations showed that crystals obtained after vapor diffusion could only be measured via synchroton radiation [31].

### Biological studies: cytotoxicity

**Fig. 9:**
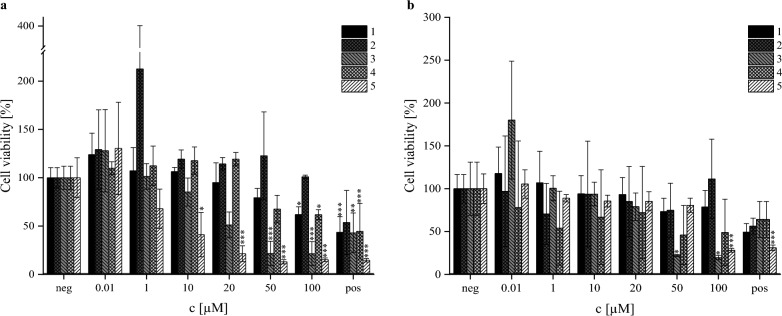
Cytotoxicity of the isolated apicidins (**1–4**) and apicidin (**5**) via resazurin assay in HepG2 (**a**, left) and A549 (**b**, right) after 24 h of substance incubation normalized to the negative control (neg, 1% DMSO). Applied substance concentration ranged from 0.01 to 100 µM. Positive control (pos) was 10 µM T2-toxin. Black shows apicidin F (**1**), dark grey shows apicidin J (**2**), grey shows apicidin K (**3**), light grey shows apicidin L (**4**), and white shows apicidin (**5**). Experiments were conducted with three individual cell lines with three biological replicates each (*N* = 9). Columns depict mean values ± standard deviations in %. Statistically significance was tested by one-way ANOVA and post hoc Tukey’s test: *p ≤ 0.05; **p ≤ 0.01; ***p ≤ 0.001

In previous studies, the cytotoxicity of apicidin F (**1**) was investigated in comparison to apicidin (**5**), and it was shown that the derivative has a significantly less toxic effect on human liver carcinoma cells (HepG2) via the CCK-8 assay [21]. In this study, the effect on cellular viability was examined for all four derivatives (**1–4**) and apicidin (**5**) on HepG2 (HB-8065) and human lung adenocarcinoma cells (A549, CCL-185) in a concentration range of 0.01–100 µM via the resazurin assay. The cell viability of both cell lines after this investigation is depicted in Fig. 9.

In general, all apicidin-derivates show lower cytotoxic effects than apicidin (**5**). With regard to apicidin (**5**) and apicidin F (**1**), the results are in alignment with previous investigations [21], with a IC_50_ observed at 2.7 µM (apicidin, **5**) and no observed IC_50_ for apicidin F (**1**) in the tested concentration range. Similarly, due to low cytotoxic effects of apicidin J (**2**) and apicidin L (**4**), no IC_50_ could be calculated in neither cell line. In contrast, cytotoxic effects of apicidin K (**3**) were observed in both cell lines, yielding an IC_50_ = 17.0 µM (HepG2) and IC_50_ = 25.5 µM (A549). It is postulated that the compounds carrying d-proline (apicidin J, **2**; apicidin L, **4**) experience a detoxification due to the substitution of d-pipecolic acid, while the modification of the l-2-aminooctanedioic acid-sidechain seems to cause a toxification of the substance, as it is observed for apicidin K (**3**). In case of apicidin L (**4**), it might be possible that the detoxification effect from d-proline is able to overcome the toxification observed from the presence of l-2-amino-8-hydroxyoctanoic acid within the tested concentration range.

The evaluation of the cytotoxicity of the isolated compounds against the L6 cell line (rat myoblast) showed a similar tendency, whereas IC_50_ for apicidin K (**3**) was calculated to be 7.2 µM. Apicidin F (**1**), among others, was found to be less toxic with an IC_50_ value of 63.7 µM. Newly isolated apicidin L (**4**) and apicidin J (**2**) demonstrated moderate cytotoxic effects with IC_50_ = 20 µM and IC_50_ = 46.5 µM, respectively.

### Antiparasitic activity

When apicidin (**5**) was first characterized by Singh et al. in 1996 [32], it was also investigated for its antiparasitic activity in vivo against *Plasmodium berghei* and in vitro against *Plasmodium falciparum*, both are transmitters of malaria tropica [14]. Indeed, an effect was observed, which was later-on discovered to be exerted from reversible inhibition of apicomplexan HDACs [14, 15]. In case of apicidin F (**1**), an IC_50_ = 0.67 µM was observed against *P. falciparum*, without further investigating the potential mode of action [22]. Therefore, it was reasoned to also unravel the biological activity of all four purified substances.

Interestingly, all isolated apicidins (**1**–**4**) demonstrated no activity against *Trypanosoma brucei rhodesiense* STIB 900. In contrast, they were found to be toxic when incubated with *P. falciparum* NF54. Apicidin F (**1**) demonstrated the highest inhibition of *P. falciparum* growth with IC_50_ = 0.51 µM, which corroborated with previously published data [22]. Apicidins J (**2**) and K (**3**) showed equal activity, as the IC_50_ for both compounds were calculated to be 1.1 µM. Apicidin L (**4**) was found to be less active among screened compounds (**1–4**) with IC_50_ = 2.1 µM.

### In vitro inhibition of selected HDAC isoforms

Additionally, the potential suitability of all apicidins toward HDAC inhibition was examined, the results are summarized in Table 2. Initially, apicidins F (**1**), J (**2**), K (**3**), and L (**4**) were tested concerning their ability to inhibit HDAC1 and 6 using vorinostat as reference compound. Additionally, apicidins F (**1**) and J (**2**) were tested against HDAC2 and 3. Both apicidins bearing l-2-amino-8-hydroxyoctanoic acid in their amino acid sequence (apicidins K (**3**) and L (**4**)) showed no HDAC1 and HDAC6 inhibition up to 3.33 µM or 10 µM, respectively. In contrast, apicidins F (**1**) and J (**2**) bearing l-2-aminooctanedioic acid in their amino acid sequence showed moderate submicromolar inhibition of the class I isoforms HDAC1-3 and single-digit micromolar inhibition at HDAC6. Furthermore, and in good agreement with the results of the cytotoxicity screening, the d-pipecolic acid derivative apicidin F (**1**) showed vorinostat-like HDAC1/2 inhibition and emerged as the most potent HDACi of the evaluated apicidins (HDAC1 IC_50_ = 0.102 μM; HDAC2 IC_50_ = 0.164 μM; HDAC3 IC_50_ = 0.202 μM). In contrast, the d-proline derivative apicidin J (**2**) showed less potent HDAC1-3 inhibition with IC_50_ values ranging from 0.342 µM (HDAC1) to 0.754 µM (HDAC3).

**Table 2:** Inhibitory activity of apicidins against HDAC1-3 and 6


Code	n	R^1^	IC_50_ [µM]			
			HDAC1 ^[a]^	HDAC2 ^[a]^	HDAC3 ^[a]^	HDAC6 ^[a]^
**Apicidin F (1)**	**2**		0.102 ± 0.004	0.164 ± 0.017	0.202 ± 0.026	6.38 ± 1.57
**Apicidin J (2)**	**1**		0.342 ± 0.018	0.633 ± 0.028	0.754 ± 0.112	9.87 ± 1.40
**Apicidin K (3)**	**2**		> 3.33 ^[b]^	n.d.	n.d.	> 10 ^[b]^
**Apicidin L (4)**	**1**		> 10 ^[b]^	n.d.	n.d.	> 10 ^[b]^
**Vorinostat**	–	0.116 ± 0.012	0.183 ± 0.008	0.095 ± 0.006	0.038 ± 0.008	

[a] mean ± standard deviation (SD) of at least two independent experiments; [b] < 30% inhibition at the stated concentration; n.d.: not determined

## Conclusion

In conclusion, the family of apicidins was extended by a newly characterized compound: apicidin L (**4**). It is a 12-membered cyclic tetrapeptide and consists of *N*-methoxy-l-tryptophane, d-proline, l-2-amino-8-hydroxyoctanoic acid, and l-phenylalanine in order of their sequence. According to IUPAC, its chemical annotation is (3*S*,6*S*,9*S*,14a*R*)-9-benzyl-3-(6-hydroxyhexyl)-6-[(1-methoxy-1H-indol-3-yl)methyl]decahydropyrrolo[1,2-a][1,4,7,10]tetraazacyclododecine-1,4,7,10-tetraone. Furthermore, its cytotoxic effects were tested in three different cell lines, exhibiting cytotoxic effects only at high concentrations in rat myoblast cells (IC_50_ = 20 µM). Regarding its antimalarial activity, no effect against *Trypanosoma brucei rhodesiense* was observed, while an IC_50_ = 2.1 µM determined for in vitro activity against *Plasmodium falciparum*. Furthermore, no HDAC inhibition of HDAC1, 2, 3, or 6 could be determined for this substance.

Alongside apicidin L (**4**), three more apicidins derived from genetically modified strains of *Fusarium fujikuroi* have been tested. All of them exerted cytotoxic effects on the L6 cell line and none showed toxic effect in vitro against *T.b. rhodesiense*. However, apicidin F (**1**) showed highest antimalarial activity against *P. falciparum* at an IC_50_ = 0.51 µM, while apicidins K (**3**) and J (**2**) had similar inhibitory values (both IC_50_ = 1.1 µM). With regard to the mode of action, apicidin F (**1**) showed a comparable IC_50_ value for HDAC1 inhibition as the positive control (0.102 ± 0.004 µM and 0.116 ± 0.012 µM, respectively). Overall, apicidin F (**1**) was the most active substance tested for all four HDACs. Apicidin J (**2**) also exhibited activity toward all tested HDACs, however, presented higher IC_50_ values compared to apicidin F (**1**).

## Experimental section

### General experimental procedures

Instrumental screening of all samples was done with HPLC-HRMS. Liquid chromatographic separation was achieved with a ReproSilGold C_18_-AQ column (150 × 2 mm i.d., 3 µm) equipped with a 5 × 2 mm i.d. ReproSilPur C_18_-AQ, 3 µm guard column (both Dr. Maisch HPLC GmbH, Ammerbuch, Germany) on a Shimadzu HPLC system (DGU-20A5R degasser; Nexera XT LC-20AD XR pump; Nexera XR, SIL-20AC XR autosampler, CTO-10SD VP oven unit; connected through a CBM-20A communication bus module, all Shimadzu, Tokyo, Japan), coupled to a high resolution Fourier transform mass spectrometer with a heated electrospray ionization source (LTQ Orbitrap XL, Thermo Scientific, Dreieich, Germany). The solvents used for LC separation were H_2_O + 0.1% FA (solvent A) and MeCN + 0.1% FA (solvent B). The gradient had a flow rate of 0.3 mL/min and started at 10% B for 1 min, was increased to 100% B over 20 min, held at 100% for additional 5 min, dropped to starting conditions within 0.1 min, and re-equilibrated for 4.9 min. The oven temperature was set to 40.0 °C. For ionization, HESI was applied. Capillary and vaporizer temperature were set to 350 °C each. Gas flows were 40 arbitrary units (AU) for sheath gas, and 20 AU and 5 AU for auxiliary gas flow and sweep gas flow, respectively. The source voltage was 3.5 kV, source current was 100 µA, capillary voltage was at 20 V and tube lens voltage at 120 V. The untargeted screening measurement ranged from *m/z* 150-1000. Data acquisition and evaluation was performed using the Xcalibur Tune Plus software, Version 3.1.66.10 (Thermo Fisher Scientific, Dreieich, Germany).

Furthermore, general screening was performed with TLC. For each analyte, a standard solution of 200 µg/mL in 80/20 MeCN/H_2_O (v/v) was prepared using pure substance. For chromatographic separation, 10  µL of the standard solution was applied to aluminum plates (5 × 10 cm) modified with RP-18 W nano silica gel with a fluorescent indicator at λ = 254 nm with a thin glass capillary. The mobile phase consisted of 30/70 MeCN/H_2_O (v/v), and the plates were developed for 10 min. Determination of the retention factors (R_f_) was performed under UV-light at λ = 254 nm by dividing the distance travelled by the analyte by the distance of the solvent front. The R_f_ values were R_f_ = 0.41 (apicidin F, **1**), R_f_ = 0.49 (apicidin J,** 2**), R_f_ = 0.10 (apicidin K, **3**), and R_f_ = 0.21 (apicidin L, **4**).

### Chemicals

All solvents and reagents were purchased from Sigma-Aldrich (Deisenhofen, Germany), VWR (Darmstadt, Germany), Fisher Scientific Inc. (Schwerte, Germany), or Merck Schuchardt (Hohenbrunn, Germany) in gradient or analytical grade. Water for chromatography was purified with a PURELAB Flex 2 system (Veolia Water Technologies, Celle, Germany). d/l-2-aminooctanoic acid, l-glutamic acid, l-tryptophane, polyethylene glycol 3350, and resazurin sodium salt were purchased from Sigma-Aldrich/Merck KGaA (Darmstadt, Germany). l-lysin-monohydrate was purchased from Biosynth® GmbH (Berlin, Germany), l-phenylalanine was purchased from Flunka™ Biochemica, Honeywell Inc., (Offenbach, Germany). Copper sulfate pentahydrate, zinc sulfate heptahydrate, dimethylsulfoxid, and 2-[4-(2-hydroxyethyl)piper-azin-1-yl]ethanesulfonic acid were purchased from Carl Roth GmbH + Co. KG (Karlsruhe, Germany). Fetal calf serum, penicillium/streptomycin solution, and phosphate bovine serum was purchased from PAN™ Biotech (Aidenbach, Germany). Trypsin was purchased from Merck Biochrom GmbH (Berlin, Germany), Dulbecco’s modified eagle medium was purchased from Thermo Fisher Gibco (Darmstadt, Germany). Ethyl acetate and ammonium sulfate were purchased from Grüssing GmbH (Filsum, Germany).

### Fungal material

The filamentous fungus *Fusarium fujikuroi* IMI58289 (Commonwealth Mycological Institute, Kew, UK) and the mutants Δ*APF1*, OE::*APF2*, Δ*APF3/*OE::*APF2*, and Δ*APF9/*OE::*APF2* were used for the investigations [20]. For pre-cultivation, 300-mL Erlenmeyer flasks containing 100 mL Darken’s medium (DVK) [33] were inoculated with a 1 cm^2^ agar plug of the respective strain and shaken at 28 °C at 180 rpm for three days in the dark (New Brunswick Innova® 44 incubator shaker, Eppendorf, Hamburg, Germany). Standard culture was grown by inoculating each DVK agar plates with 500 µL of the liquid DVK pre-culture and cultivate for 5–10 days in an incubator (KB-115 refrigerated incubator, BINDER GmbH, Tuttlingen, Germany) at 28 °C and saturated humidity in the dark.

### Micro-scale cultivation

For fungal cultivation, sterile flat bottom 96-well plates (Sarstedt AG & Co.KG, Nümbrecht, Germany) were prepared by adding 150 µL of medium with a Multipette® M4 equipped with a sterile 5 mL Combitip® advanced (both Eppendorf SE, Hamburg, Germany) to each well. Upon solidification of the media, the 96-well plate was inoculated with 50 µL of liquid pre-culture and grown for 5 days in the incubator at 28 °C in the dark. Extraction was carried out twice with MeOH/H_2_O (80/20, v/v). Here, 100 µL of extraction solution were added to the culture and agitated with the pipette tip to separate the mycelium from the agar and disrupt the fungal material. Then, the solution was transferred to a MULTI 96 filterplate (CHROMAFIL® PTFE, 8 mm, 0.45 µm pore size, Macherey–Nagel, Düren, Germany). This extraction step was repeated a second time before the filter was transferred to a NucleoVac 96 Vacuum Manifold (Macherey–Nagel, Düren, Germany) containing a Nunc™ 96-well polypropylene sample processing & storage microplate (Thermo Fisher Scientific Inc., Darmstadt, Germany). The samples were filtered under reduced pressure, the sample plate was covered with ThinSeal™ sealing films (EXCEL Scientific, Victorville, CA, USA) and submitted for HPLC-HRMS measurement.

### Supplementation screening

For supplementation screening, the fungal strains were grown in 96-well plates containing solid DVK agar plugs as previously described. The supplementation was performed with single as well as combined amino acids. A control row without any supplementation was always included. For the initial experiments, low (5 mM) and high (50 mM) amino acid concentrations were prepared. This was done by weighting the respective amino acid on an analytical scale (Acculab Atilon Analytical Balance, Sartorius AG, Göttingen, Germany) and dissolving the powder into the hand-warm medium after autoclavation. For the optimization of the 2-aoa supplementation, standard agar plates were cast, and the fungal cultivation followed the standard procedure as presented previously.

### Laboratory-scale supplemented culture and compound isolation procedure

For laboratory-scale cultivation of both strains, D/L-2-aminooctanoic acid was supplemented to the DVK medium at a final concentration of 20 mM. For the OE::*APF2* strain, 150 supplemented agar plates were cast, while for Δ*APF9*/OE::*APF2*, 150 standard agar plates and 200 supplemented agar plates were prepared and each inoculated with 500 µL of liquid DVK pre-culture. Cultivation was performed in the incubator for 5 days at 28 °C in the dark. Then, agar plates were scraped into a 5 L container and extracted three times with 2.5 L EtOAc. The extract was evaporated to dryness on a rotary evaporator at 40 °C (Laborota 4000 rotary evaporator, Heidolph, Schwabach, Germany, connected to a Duo Chiller RC-10 cooling system, VWR®, Darmstadt, Germany, with a PC510 vacuum pump equipped with a CVC 2 vacuum controller, both Vacuubrand™, Wertheim, Germany), remaining water was removed by lyophilization (Lyovac GT 2, Finn-Aqua Sanatasalo-Sohlberg GmbH, Cologne, Germany). The crude extract was re-constituted with 4 mL MeCN/H_2_O (80/20, v/v) and applied to a 5 g/20 mL Strata® C_18_-E (55 µm, 70 Å) SPE column (Phenomenex, Aschaffenburg, Germany). In detail, the SPE was conditioned with 50 mL MeCN, and equilibrated with 50 mL H_2_O before loading the sample. Here, after loading 1 mL of sample, 10 mL of H_2_O were applied as an additional washing step. Elution was performed by applying a gradient of MeCN and H_2_O which increased the ratio of MeCN in steps of 10% until 50/50 MeCN/H_2_O (v/v), followed by an elution with 100% MeCN. Elution was accelerated by application of reduced pressure (400 mbar) to the outlet of the SPE-chamber. The collected fractions were analyzed by TLC and HPLC-HRMS to identify the analyte-containing fractions, which were combined and once again brought to dryness as previously described. Next, the dried fractions were re-constituted in 4 mL of 80/20 MeCN/H_2_O (v/v) and further purified on a preparative HPLC–UV system (Degas® GPC degasser, Biotech I Kungsbacka AB, Onsala, Sweden, Jasco PU-2087 pump coupled to Jasco UV-2075-detector equipped with a Jasco AS-2057 Plus direct injection system interfaced by a Jasco LC-Net II / ADC, Jasco, Groß-Umstadt, Germany, and a Column Thermostat Jetstream 2 oven, VDS optilap Chromatographietechnik GmbH, Berlin, Germany). The chromatographic separation was achieved on a ReprosilPur 120 C_18_ (250 × 10 mm, 5 µm) semi-preparative column (Dr. Maisch GmbH, Ammerbuch, Germany) equipped with a SecurityGuard™ Gemini® C_6_-phenyl cartridge (4 × 2 mm) guard column (Phenomenex, Aschaffenburg, Germany) and a gradient consisting of H_2_O (solvent A) and MeCN (solvent B). The gradient started with 10% B, was held for 2 min, raised to 60% B within 11.5 min, held until 16 min, increased to 100% within 3 min, held for another 4 min, changed back to starting conditions within 0.01 min and re-equilibrated for 5 min. Each chromatographic run was monitored with a UV-detector set to λ = 225 nm, flow rate was set to 5 mL/min, oven temperature was at 40 °C. Data evaluation was done using ChromPass Software (version 1.8.6.1, Jasco, Groß-Umstadt, Germany). Apicidin F (**1**) and K (**3**) eluted after 15.4 and 15.2 min, and apicidin J (**2**) and L (**4**) eluted at 13.7 and 13.6 min. The compound purity was determined on a HPLC–DAD-ELSD system (Jasco PU-2087 pump coupled to a MD-2010 Plus diode array detector interfaced by a LC-Net II/ADC, Jasco, Groß-Umstadt, Germany, with a Column Thermostat Jetstream 2 oven, VDS optilap Chromatographietechnik GmbH, Berlin, Germany and an evaporative light scattering detector, Shimadzu, Tokyo, Japan), using a ReprosilGold C_18_-AQ column (150 × 2 mm i.d., 3 µm) equipped with a 5 × 2 mm i.d. guard column of the same material (both Dr. Maisch HPLC GmbH, Ammerbuch, Germany). The 30 min gradient was performed with H_2_O + 0.1% FA (solvent A) and MeCN + 0.1% FA (solvent B). The oven temperature was set to 40 °C, flow rate was at 0.3 mL/min. The gradient started at 10% B for one minute, was increased to 100% B over 20 min, held at 100% for additional 5 min, dropped to starting conditions within 0.1 min, and re-equilibrated for 4.9 min. The DAD spectrum was recorded between 195 and 650 nm, ELSD was set to 50 °C at 350 kPa with compressed air, and the gain was 10. The isolated compounds were analyzed at a concentration of 200–400 µg/mL in 80/20 MeCN/H_2_O (v/v). Their purity was determined by comparing 20 µL injection of the analyte solution with a previously injected 80/20 MeCN/H_2_O (v/v) blank solution. Here, the injection peak and solvent signals from the gradient were excluded from the evaluation. Data evaluation was done using ChromPass Software (version 1.8.6.1, Jasco, Groß-Umstadt, Germany).

The final yield of each compound (isolated with and without supplementation with dl-2-aminooctanoic acid) was 7.5 mg of apicidin F (**1**) at ≥ 98% purity from 150 supplemented agar plates, 7.9 mg of apicidin J (**2**) at ≥ 98% purity from 150 supplemented agar plates, 13.3 mg of apicidin K (**3**) at ≥ 98% purity from 200 supplemented and 150 standard agar plates, and 25.2 mg of apicidin L (**4**) at ≥ 98% purity from 200 supplemented and 150 standard agar plates.

### Structure elucidation of apicidin L

The structure elucidation was performed using NMR-spectroscopy and HRMS fragmentation experiments. The ^1^H, ^13^C, and 2D NMR spectra were recorded on a DD2 600 MHz NMR spectrometer (Agilent Technologies, Santa Clara, CA, USA). The signals are reported in parts per million (ppm) and are referenced to the solvent. For structure elucidation, additionally to the 1D NMR experiments, 2D NMR experiments such as H,H-correlated spectroscopy (H,H-COSY), heteronuclear multiple-quantum correlation (HMQC), heteronuclear multiple bond correlation (HMBC), and rotating Overhauser enhancement spectroscopy (ROESY) were performed. The pulse programs were taken from the software library. NMR measurements were performed with apicidin L (**4**) dissolved in C_5_D_5_N to show allocation of nitrogen-coupled protons. -, ^1^ The obtained data was evaluated using MestReNova 14.2 (Mestrelab Research S.L., Santiago de Compostela, Spain). The complete dataset can be found in the Additional file 1.

The HRMS fragmentation experiments were performed on a Thermo Scientific LTQ Orbitrap XL system (Thermo Scientific, Dreieich, Germany) using direct injection with a syringe pump. apicidin L (**4**) was dissolved in 80/20 MeCN/H_2_O (v/v) containing 1% acetic acid at a concentration of 15 µg/mL. The injection flow rate was 5 µL/min. The ionization was performed in positive mode with a heated electrospray ionization (HESI) applying the following parameters: sheath gas flow was at 5 AU, aux gas flow and sweep gas flow were not applied. The HESI was turned off. Capillary temperature was set to 275 °C, while source voltage was at 4.0 kV, capillary voltage was at 43 V and tube lens voltage was set to 150 V. Fragmentation experiments were performed with collision-induced dissociation (CID) at an isolation width of 1.8 Da. For MS^2^, the relative fragmentation energy was at 11%, while for MS^3^ and MS^4^ 10% to 13% were applied depending on the parent ion to be fragmented. Data evaluation was performed using the Xcalibur software (version 3.1, Thermo Fisher Scientific, Dreieich, Germany).

**Apicidin F (1)** Yellow-white powder (7.5 mg from 150 agar plates). TLC: 0.41 (MeCN/H_2_O, 30/70, v/v). UV (MeCN/H_2_O + 0.1% FA) λ_max_ (log ε): 230, 290 nm. ^1^H NMR (CD_3_OD, 600 MHz) δ 7.57 (1H, d, *J* = 7.9 Hz, H-5), 7.43 (2H, d, *J* = 18.8 Hz, H-5‴, H-9‴), 7.36 (1H, d, *J* = 8.2 Hz, H-8), 7.32 (2H, s, H-6‴, H-8‴), 7.29–7.23 (1H, m, H-7), 7.23–7.15 (1H, m, H-7‴), 7.06 (1H, t, *J* = 7.5 Hz, H-6), 7.02 (1H, s, H-9), 5.26 (1H, t, *J* = 7.7 Hz, H-2‴), 5.17 (1H, d, *J* = 5.8 Hz, H-2″), 4.61 (1H, d, *J* = 16.1 Hz, H-2), 4.30 (1H, dt, *J* = 15.6, 8.1 Hz, H-2′), 4.00 (3H, s, H-10), 3.81 (2H, d, *J* = 13.5 Hz, H-6″), 3.28–3.16 (4H, m, H-3, H-3‴), 3.04 (2H, dd, *J* = 13.5, 7.5 Hz, H-3‴), 2.93 (2H, t, *J* = 13.3 Hz, H-6″), 2.19 (2H, t, *J* = 7.6 Hz, H-7′), 1.96 (2H, d, *J* = 14.5 Hz, H-3″), 1.72–1.66 (2H, m, H-3′), 1.62 (2H, d, *J* = 12.2 Hz, H-4′), 1.56 (2H, s, H-6′), 1.49 (3H, d, *J* = 15.6 Hz, H-2″, H4″), 1.44 (2H, dt, *J* = 12.9, 5.6 Hz, H-3″), 1.36–1.25 (2H, m, H-5′), 1.17 (2H, dd, *J* = 30.7, 11.8 Hz, H-5″). ^13^C NMR (CD_3_OD, 150 MHz) δ 175.87 (C, C-1′), 175.44 (C, C-1), 175.00 (C, C-1‴), 173.49 (C, C-1″), 138.49 (C, C-4‴), 133.86 (C, C-8a), 130.42 (CH, C-5‴, C-9‴), 129.60 (CH, C-6‴, C-8‴), 127.89 (CH, C-7‴), 125.01 (C, C-4a), 123.62 (CH, C-7), 123.04 (CH, C-9), 120.88 (CH, C-6), 119.74 (CH, C-5), 109.34 (CH, C-8), 107.72 (C, C-4), 66.21 (CH_3_, C-10), 59.43 (CH, C-2), 56.51 (CH, C-2′), 52.12 (CH, C-2″), 51.45 (CH, C-2‴), 45.20 (CH_2_, C-6″), 37.81 (CH_2_, C-3‴), 30.68 (CH_2_, C-3′), 29.86 (CH_2_, C-5′), 26.85 (CH_2_, C-4′), 26.68 (CH_2_, C-3), 26.40 (CH_2_, C-5″), 26.21 (CH_2_, C-6′), 25.23 (CH_2_, C-3″), 20.62 (CH_2_, C-4″). Positive ion HRESIMS (25 µg/mL): *m/z* 646.3230 (calcd for C_35_H_43_N_5_O_7_ [M + H]^+^ 646.3236, Δm: 0.9 ppm), purity ≥ 98%.

**Apicidin J (2)** White powder (7.9 mg from 150 agar plates). TLC: 0.49 (MeCN/H_2_O, 30/70, v/v). UV (MeCN/H_2_O + 0.1% FA) λ_max_ (log ε): 220, 291 nm. ^1^H NMR (C_5_D_5_N, 600 MHz) δ 9.71 (1H, d, *J* = 7.0 Hz, NH), 8.57 (1H, d, *J* = 10.0 Hz, NH‴), 7.89 (1H, d, *J* = 10.0 Hz, NH′), 7.75 (1H, d, *J* = 7.9 Hz, H-5), 7.53 (1H, d, *J* = 8.2 Hz, H-8), 7.46 (3H, dd, *J* = 9.9, 2.8 Hz, H-5‴, H-9 H-9‴), 7.34 (3H, dt, *J* = 10.8, 7.5 Hz, H-7, H-6‴, H-8‴), 7.30–7.23 (2H, m, H-7‴), 7.17 (1H, t, *J* = 7.4 Hz, H-6), 5.58 (1H, dd, *J* = 8.7, 7.0 Hz, H-2‴), 5.26 (1H, s, OH′), 5.00 (1H, dd, *J* = 7.8, 1.5 Hz, H-2″), 4.78 (1H, t, *J* = 7.8 Hz, H-2′), 4.69–4.62 (1H, m, H-2), 4.08 (2H, ddd, *J* = 20.2, 13.9, 9.3 Hz, H-3, H-5″), 3.93 (3H, s, H-10), 3.89–3.81 (3H, m, H-3), 3.55 (4H, dd, *J* = 13.6, 8.6 Hz, H-3‴), 3.37–3.33 (2H, m, H-5″), 3.30 (2H, dd, *J* = 13.6, 7.0 Hz, H-3‴), 2.42 (2H, t, *J* = 7.5 Hz, H-7′), 2.39–2.32 (2H, m, H-3″), 2.10 (2H, ddd, *J* = 19.8, 11.9, 8.9 Hz, H-4″), 2.06–1.94 (4H, m, H-3′, H-6′), 1.74–1.66 (4H m,, H-3′, H-6′), 1.57 (2H, dtd, *J* = 12.2, 8.0, 4.0 Hz, H-4″), 1.47–1.37 (2H, m, H-3″), 1.36–1.26 (4H, m, H-4′, H-5′). ^13^C NMR (C_5_D_5_N, 150 MHz) δ 176.07 (C, C-1′), 175.23 (C, C-1), 174.04 (C, C-1‴), 172.48 (C, C-1″), 138.66 (C, C-4‴), 133.51 (C, C-8a), 130.27 (CH, C-5‴, C-9‴), 129.54 (CH, C-6‴, C-8‴), 127.68 (CH, C-7‴), 124.81 (C, C-4a), 123.47 (CH, C-7), 123.29 (CH, C-9), 120.79 (CH, C-6), 120.03 (CH, C-5), 109.44 (CH, C-8), 108.65 (C, C-4), 66.30 (CH_3_, C-10), 61.59 (CH, C-2), 58.75 (CH, C-2″), 56.04 (CH, C-2′), 54.36 (CH, C-2‴), 47.31 (CH_2_, C-5″), 37.17 (CH_2_, C-3‴), 35.30 (CH_2_, C-7′), 30.83 (CH_2_, C-3′), 29.68 (CH_2_, C-5′), 26.56 (CH_2_, C-4′), 26.48 (CH_2_, C-6′), 25.89 (CH_2_, C-3), 25.63 (CH_2_, C-3″), 25.35 (CH_2_, C-4″). Positive ion HRESIMS (25 µg/mL): *m/z* 632.3071 (calcd for C_34_H_41_N_5_O_7_ [M + H]^+^ 632.3079, Δm: 1.3 ppm), purity ≥ 98%.

**Apicidin K (3)** White powder (13.3 mg from 200 supplemented and 150 standard agar plates). TLC: 0.10 (MeCN/H_2_O, 30/70, v/v). UV (MeCN/H_2_O + 0.1% FA) λ_max_ (log ε): 235, 291 nm. ^1^H NMR (C_5_D_5_N, 600 MHz) δ 9.97 (1H, d, *J* = 6.9 Hz, NH), 8.55 (1H, d, *J* = 10.1 Hz, NH‴), 7.73 (1H, d, *J* = 8.0 Hz, H-5), 7.54 (1H, d, *J* = 8.2 Hz, H-8), 7.47 (2H, d, *J* = 7.2 Hz, H-5‴, H-9‴), 7.42 (1H, s, H-9), 7.33 (3H, q, *J* = 7.3 Hz, H-7, H-6‴, H-8‴), 7.30–7.25 (1H, m, H-7‴), 7.17 (1H, t, *J* = 7.5 Hz, H-6), 5.85 (1H, dt, *J* = 10.1, 7.5 Hz, H-2‴), 5.48 (1H, d, *J* = 5.7 Hz, H-2″), 5.16 (1H, s, OH′), 4.79 (1H, q, *J* = 8.6 Hz, H-2′), 4.55 (1H, dt, *J* = 9.8, 6.7 Hz, H-2), 4.33 (1H, d, *J* = 13.3 Hz, H-6″), 4.15 (2H, dd, *J* = 14.5, 9.9 Hz, H-3), 3.92 (3H, s, H-10), 3.80 (2H, t, *J* = 6.5 Hz, H-8′), 3.76 (2H, dd, *J* = 14.5, 6.7 Hz, H-3), 3.55 (2H, dd, *J* = 13.8, 7.3 Hz, H-3‴), 3.40 (2H, dd, *J* = 13.8, 7.6 Hz, H-3‴), 3.30–3.22 (2H, m, H-6″), 2.28 (2H, dd, *J* = 11.2, 6.7 Hz, H-4″), 2.01 (2H, d, *J* = 13.4 Hz, H-3″), 1.95 (2H, dt, *J* = 18.0, 7.1 Hz, H-3′), 1.62 (4H, p, *J* = 7.1 Hz, H-3′, H-7′), 1.42–1.28 (8H, m, H-3″, H-4″, H-5″, H-6′), 1.28–1.18 (4H, m, H-4′, H-5′), 1.09 (2H, tdd, *J* = 13.2, 9.3, 4.0 Hz, H-5″). ^13^C NMR (C_5_D_5_N, 150 MHz) δ 177.18 (C, C-1′), 174.74 (C, C-1‴), 174.36 (C, C-1), 172.53 (C, C-1″), 138.87 (C, C-4‴), 133.45 (C, C-8a), 130.26 (CH, C-5‴, C-9‴), 129.28 (CH, C-6‴, C-8‴), 127.40 (CH, C-7‴), 124.63 (C, C-4a), 123.38 (CH, C-7), 123.34 (CH, C-9), 120.70 (CH, C-6), 119.88 (CH, C-5), 109.37 (CH, C-8), 108.63 (C, C-4), 66.16 (CH_3_, C-10), 62.46 (CH_2_, C-8′), 61.96 (CH, C-2), 55.53 (CH, C-2′), 51.58 (CH, C-2″), 50.93 (CH, C-2‴), 44.75 (CH_2_, C-6″), 38.00 (CH_2_, C-3‴), 34.03 (CH_2_, C-7′), 30.85 (CH_2_, C-3′), 29.81 (CH_2_, C-5′), 26.64 (CH_2_, C-4′), 26.56 (CH_2_, C-6′), 26.04 (CH_2_, C-3), 25.02 (CH_2_, C-3″), 20.42 (CH_2_, C-4″). Positive ion HRESIMS (25 µg/mL): *m/z* 632.3432 (calcd for C_34_H_43_N_5_O_6_ [M + H]^+^ 632.3443, Δm: 1.7 ppm), purity ≥ 98%.

**Apicidin L (4)** White powder (25.2 mg from 200 supplemented and 150 standard agar plates). TLC: 0.21 (MeCN/H_2_O, 30/70, v/v). UV (MeCN/H_2_O + 0.1% FA) λ_max_ (log ε): 235, 287 nm. ^1^H NMR (C_5_D_5_N, 600 MHz) δ 9.96 (1H, d, *J* = 6.7 Hz, NH), 8.55 (1H, d, *J* = 10.1 Hz, NH‴), 7.86 (1H, d, *J* = 10.0 Hz, NH′), 7.74 (1H, d, *J* = 7.9 Hz, H-5), 7.53 (1H, d, *J* = 8.1 Hz, H-8), 7.50–7.43 (3H, m, H-9, H-5‴, H-9‴), 7.36–7.30 (3H, m, H-7, H-6‴, H-8‴), 7.25 (1H, t, *J* = 8.1 Hz, H-7‴), 7.18 (1H, t, *J* = 7.0 Hz, H-6), 5.61 (1H, ddd, *J* = 10.2, 8.7, 6.8 Hz, H-2‴), 5.10 (1H, s, OH′), 5.03–4.95 (1H, m, H-2″), 4.75 (1H, ddd, *J* = 10.0, 8.4, 6.8 Hz, H-2′), 4.48 (1H, dt, *J* = 9.6, 7.0 Hz, H-2), 4.24–4.14 (2H, m, H-3), 4.11 (2H, dd, *J* = 9.6, 3.9 Hz, H-5″), 3.91 (3H, s, H-10), 3.87 (2H, dd, *J* = 14.5, 7.3 Hz, H-3), 3.81 (2H, t, *J* = 6.5 Hz, H-8′), 3.54 (2H, dd, *J* = 13.6, 8.7 Hz, H-3‴), 3.35 (2H, dt, *J* = 10.1, 8.1 Hz, H-5″), 3.28 (2H, dd, *J* = 13.6, 6.8 Hz, H-3‴), 2.38–2.30 (2H, m, H-3″), 2.11 (2H, dt, *J* = 18.0, 6.8 Hz, H-4″), 2.06–1.89 (2H, m, H-3′), 1.66–1.60 (4H, m, H-3′, H-7′), 1.60–1.51 (2H, m, H-4″), 1.50–1.31 (4H, m, H-6′, H-3″), 1.30–1.18 (4H, m, H-4′, H-5′). ^13^C NMR (C_5_D_5_N, 150 MHz) δ 176.65 (C, C-1′), 175.13 (C, C-1), 173.85 (C, C-1‴), 172.39 (C, C-1″), 138.64 (C, C-4‴), 133.44 (C, C-8a), 130.16 (CH, C-5‴, C-9‴), 129.42 (CH, C-6‴, C-8‴), 127.53 (CH, C-7‴), 124.68 (C, C-4a), 123.36 (CH, C-7), 123.28 (CH, C-9), 120.69 (CH, C-6), 119.93 (CH, C-5), 109.35 (CH, C-8), 108.72 (C, C-4), 66.15 (CH_3_, C-10), 62.46 (CH, C-8′), 62.40 (CH_2_, C-2), 58.63, (CH, C-2″), 55.80 (CH, C-2′), 54.28 (CH, C-2‴), 47.18 (CH_2_, C-5″), 37.15, (CH_2_, C-3‴), 34.05 (CH_2_, C-7′), 30.87 (CH_2_, C-3′) 29.86 (CH_2_, C-5′), 26.67 (CH_2_, C-4′), 26.58 (CH_2_, C-6′), 26.01 (CH_2_, C-3), 25.52 (CH_2_, C-3″), 25.35 (CH_2_, C-4″). Positive ion HRESIMS (25 µg/mL): *m/z* 618.3280 (calcd for C_34_H_43_N_5_O_6_ [M + H]^+^ 618.3287, Δm: 1.1 ppm), purity ≥ 98%.

### Crystallization attempts of apicidin L

Initially, standard solutions of pure apicidin L (**4**) were prepared at a concentration of 200 µg/mL in MeCN/H_2_O. The applied attempts included slow solvent evaporation, hanging drop vapor diffusion from a 4-(2-hydroxyethyl)-1-piperazineethanesulfonic acid (HEPES) and polyethylene glycol solution, and supplementation of zinc- and copper-salts. None of these investigations resulted in formation of a single crystal but amorphous, non-defractable structures. In case of supplementation of Cu(II), a precipitate was obtained.

### Cytotoxicity assays

All four compounds (**1–4**) were taken for cytotoxicity testing and compared to apicidin (**5**). Here, two cell lines were used, human liver hepatocellular carcinoma cell line HepG2 and human lung carcinoma cell line A549. Cell viability was determined using the resazurin assay. About 1 × 10^4^ cells were seeded in 96-well plates in Dulbecco’s Modified Eagle Medium (DMEM) supplemented with 10 mM HEPES, 100 IU/mL penicillin, 100 µg/mL streptomycin, 2 mM l-glutamine and 10% (v/v) fetal calf serum (FCS) and cultivated in 5% CO_2_ at 37 °C for 24 h. After starvation for 24 h in medium without serum, the compounds of interest were applied in concentrations from 0.01 to 100 µM. The substances were incubated for 24 h. Subsequent measurement in a microplate reader (Infinite M200Pro, Tecan Austria GmbH, Männedorf, Switzerland) allowed the determination of cell viability. The data was normalized to the negative control (1% DMSO). T-2 toxin at 10 µM served as positive control. Statistical evaluation of ANOVA and post hoc Tukey’s test was performed with OriginPro 2024, version 10.1.0.170. Additionally, cytotoxicity against rat skeletal myoblasts (L6) was also determined by validated protocols at the Swiss TPH [34].

### Antiparasitic activity

Furthermore, the whole set of isolated compounds was tested in vitro against *T. brucei rhodesiense* (bloodstream trypomastigotes) and *P. falciparum* (intraerythrocytic forms). All assays were performed using validated protocols at the Swiss TPH [34].

### In vitro HDAC inhibition assay against HDAC1-3 and HDAC6

In vitro inhibitory activity against HDAC1-3 and 6 was determined using our previously published assay protocol [35, 36]. For compounds and controls, three-fold serial dilutions of the respective DMSO-stock solution in assay buffer (50 mM Tris–HCl, pH 8.0, 137 mM NaCl, 2.7 mM KCl, 1.0 mM MgCl_2_•6 H_2_O, 0.1 mg/mL BSA) were prepared and 5.0 µL of these serial dilutions were transferred into OptiPlate-96 black micro-plates (PerkinElmer). Then, 35 µL of the fluorogenic substrate ZMAL (Z-Lys(Ac)-AMC[37], 21.43 µM in assay buffer) and 10 µL enzyme solution (HDAC1-BPS Bioscience, Catalog# 50051; HDAC2-BPS Bioscience, Catalog# 50052; HDAC3/NcoR2-BPS Bioscience, Catalog# 50003; HDAC6-BPS Bioscience, Catalog# 50,006) were added. Next, the total assay volume (50 µL, max. 1% DMSO) was incubated at 37 °C for 90 min. Subsequently, 50 µL trypsin solution (0.4 mg/mL trypsin in buffer: 50 mM Tris- HCl, pH 8.0, 100 mM NaCl) was added, followed by additional 30 min of incubation at 37 °C. Fluorescence (excitation: 355 nm, emission: 460 nm) was measured using a FLUOstar OPTIMA microplate reader (BMG LABTECH). The IC_50_ values were determined by generating normalized dose–response curves using the build-in *"log(inhibitor) vs. response (three parameters)"* equation provided by GraphPad Prism (GraphPad Prism 9.0, San Diego, USA). Compounds were tested in duplicates, reported mean IC_50_ values, including standard deviation, are calculated from at least two independent experiments.

### Supplementary Information

Additional file 1: Supplementary material.

## Data Availability

All data generated and analyzed during this study are included in this published article and its Additional file 1. This includes the identification of the new apicidin L and the known apicidins by HRMS, chromatographic separation, purity chromatograms, analytical data (DAD spectra, *m/z*, NMR) of all compounds, and HRMS fragmentation spectra of apicidin L are summarized in one datafile.
