# Supplementary material for: Micro-scale screening of genetically modified Fusarium fujikuroi strain extends the apicidin family
Source: Nat Prod Bioprospect. 2024 Aug 23;14(1):51. doi: 10.1007/s13659-024-00473-9 (PMC11343938; doi:10.1007/s13659-024-00473-9)
Supplement: Supplementary file 1 — Additional file 1: Supplementary material. [file 13659_2024_473_MOESM1_ESM.pdf]

Supporting Information

Micro-scale screening of genetically modified *Fusarium fujikuroi* strain extends the apicidin family

Alica Fischle[1,2‡], Mika Lutsch[1‡], Florian Hübner[1], Linda Schäker-Hübner[3], Lina Schürmann[1], Finn K. Hansen[3], Svetlana A. Kalinina[1,2*]

[*]corresponding author; [‡]authors contributed equally

Corresponding Author

*Svetlana A. Kalinina, − *Institut für Lebensmittelchemie, Universität Münster, 48149 Münster, Germany*; orcid.org/0000-0001-7564-8213; Phone: +49 251-8333392; Email: s_kali03@uni-muenster.de; Fax: +49 251-8333396

[‡] A.F. and M.L. contributed equally to this work.

Figure S1 Extracted ion chromatograms with respective full scan of all apicidins ....……...3
Figure S2 Purity chromatograms of all apicidins………………………………………………....4
Figure S3 UV-spectra of all apicidins………………………………………………………….....5
Figure S4 ^{1}H-NMR of apicidin F in CD_3OD……………………………………………………6
Figure S5 ^{13}C-NMR of apicidin F in CD_3OD…………………………………………………..7
Figure S6 COESY-NMR of apicidin F in CD_3OD……………………………………………..8
Figure S7 HMBC-NMR of apicidin F in CD_3OD……………………………………………...9
Figure S8 HSQC-NMR of apicidin F in CD_3OD……………………………………………..10
Figure S9 ^{1}H-NMR of apicidin J in C_5D_5N…………………………………………………11
Figure S10 ^{13}C-NMR of apicidin J in C_5D_5N………………………………………………12
Figure S11 COESY-NMR of apicidin J in C_5D_5N……………………………………………13
Figure S12 HMBC-NMR of apicidin J in C_5D_5N……………………………………………..14
Figure S13 HSQC-NMR of apicidin J in C_5D_5N………………………………………………15
Figure S14 ^{1}H-NMR of apicidin K in C_5D_5N………………………………………………16
Figure S15 ^{13}C-NMR of apicidin K in C_5D_5N………………………………………………17
Figure S16 COESY-NMR of apicidin K in C_5D_5N……………………………………………18
Figure S17 HMBC-NMR of apicidin K in C_5D_5N……………………………………………19
Figure S18 HSQC-NMR of apicidin K in C_5D_5N……………………………………………..20
Figure S19 ^{1}H-NMR of apicidin L in C_5D_5N…………………………………………………21
Figure S20 ^{13}C-NMR of apicidin L in C_5D_5N………………………………………………..22
Figure S21 COESY-NMR of apicidin L in C_5D_5N……………………………………………23
Figure S22 HMBC-NMR of apicidin L in C_5D_5N……………………………………………..24
Figure S23 HSQC-NMR of apicidin L in C_5D_5N……………………………………………..25
Figure S24 ROESY-NMR of apicidin L in C_5D_5N……………………………………………26
Equation S1 Calculation of mass error in ppm……………………………………………...26
Table S1 Overview of MS^n fragmentation experiments…………...……………………………..27
Figure S25 MS^2 spectrum of apicidin L……………………………………………………….28
Figure S26 MS^3 spectrum of apicidin L……………………………………………………….28
Figure S27 MS^4 spectrum of apicidin L……………………………………………………….29
Figure S28 MS^4 spectrum of apicidin L……………………………………………………….29

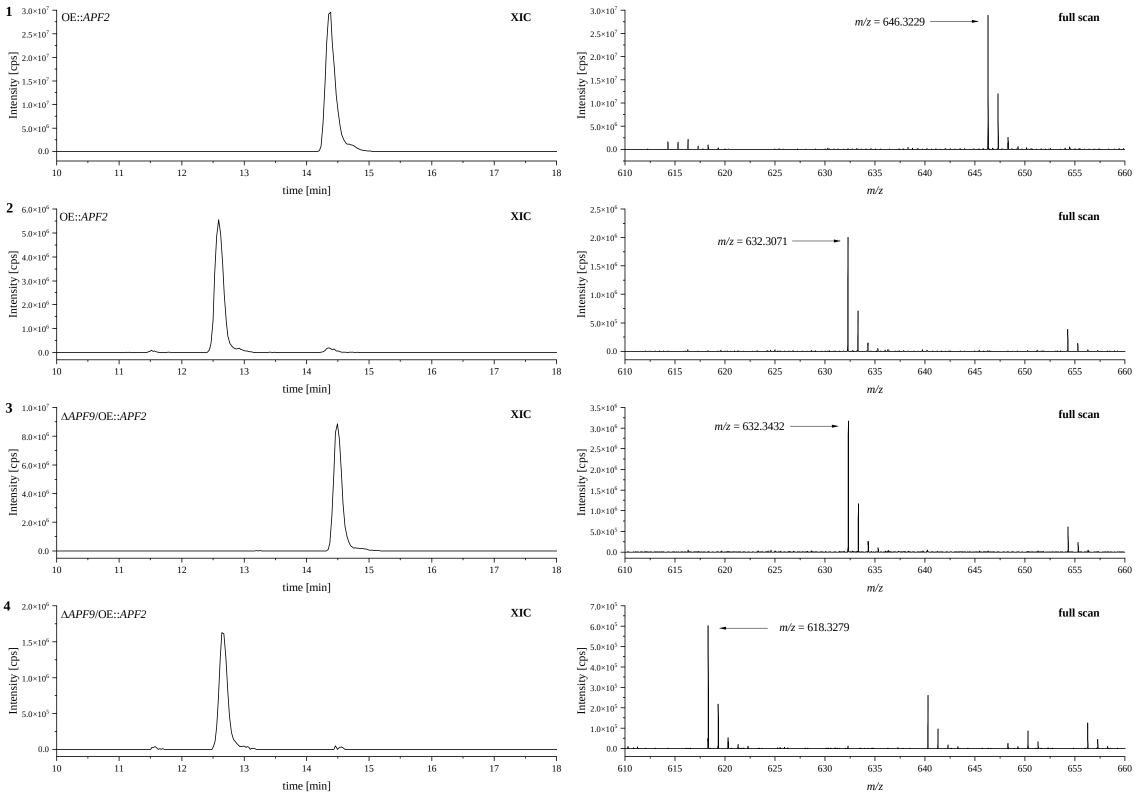

Figure S1 Extracted ion chromatograms (XIC, left) with respective full scan (right) of all apicidins. Fungal cultures were grown in 100 mL liquid Darken's medium for 14 days at 180 rpm in the dark. Apicidin F (**1**) is produced by the OE::*APF2* mutant, has a retention time of 14.4 min, and is detected at a *m/z* = 646.3229. Apicidin J (**2**) is also produced by the OE::*APF2* mutant, has a retention time of 12.6 min, and is detected at a *m/z* = 632.3071. Apicidin K (**3**) is produced by the Δ*APF9/*OE::*APF2* mutant, has a retention time of 14.5 min, and a *m/z* = 632.3432. Apicidin L (**4**) is produced by the Δ*APF9/*OE::*APF2*mutant, has a retention time of 12.7 min, and is detected at a *m/z* = 618.3279. Chromatographic separation was achieved on a ReprosilGold C_{18}-AQ (150 x 2 mm i.d., 3 µm) equipped with a 5 x 2 mm guard column of the same material. The gradient consisted of MeCN/H_2O + 0.1 % formic acid and increased linearly from 10-100% organic. Detection occurred with a LTQ Orbitrap XL in HESI positive mode with a mass accuracy of ± 2 ppm.

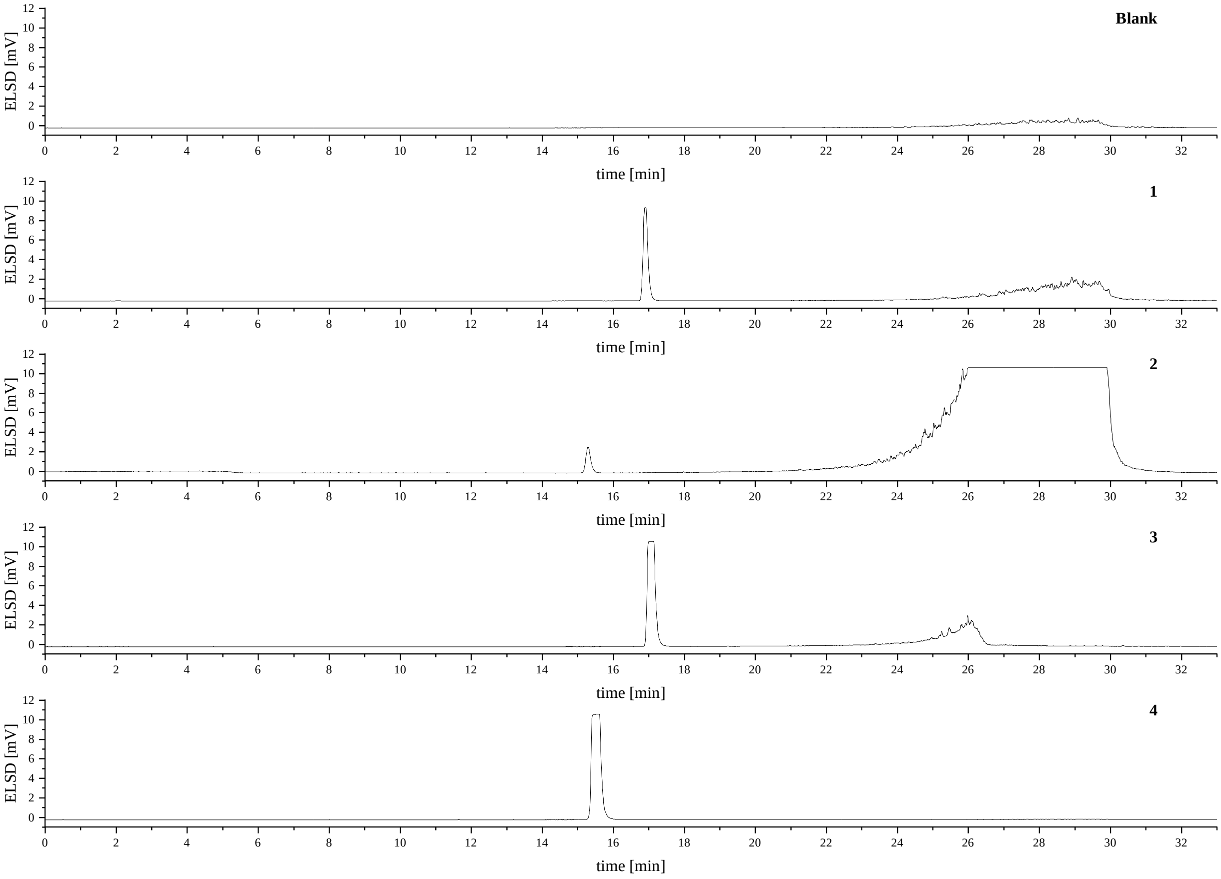

Figure S2 Purity chromatograms of all apicidins. Chromatographic separation occurred with MeCN/ H_2O + 0.1% formic acid on a ReprosilGold C_{18}-AQ column (150 x 2 mm i.d., 3 µm) equipped with a guard column of the same material (5 x 2 mm i.d.) using a linear gradient increasing from 10-100% organic phase. ELSD parameters were at 350 kPa of compressed air, 10 Gain and 50 °C. Prior to injection, a blank of 80/20 MeCN/H_2O (v/v) was measured. Apicidin F (**1**) was injected as 400 µg/mL solution, retained at 16.8 min, and showed purity ≥ 98%. Apicidin J (**2**) was injected as 200 µg/mL solution, retained at 15.2 min, and showed purity ≥ 98%. Apicidin K (**3**) was injected as 400 µg/mL solution, retained at 17.1 min, and showed purity ≥ 98%. Apicidin L (**4**) was injected as a 400 µg/mL solution, retained at 15.5 min, and showed purity ≥ 98%.

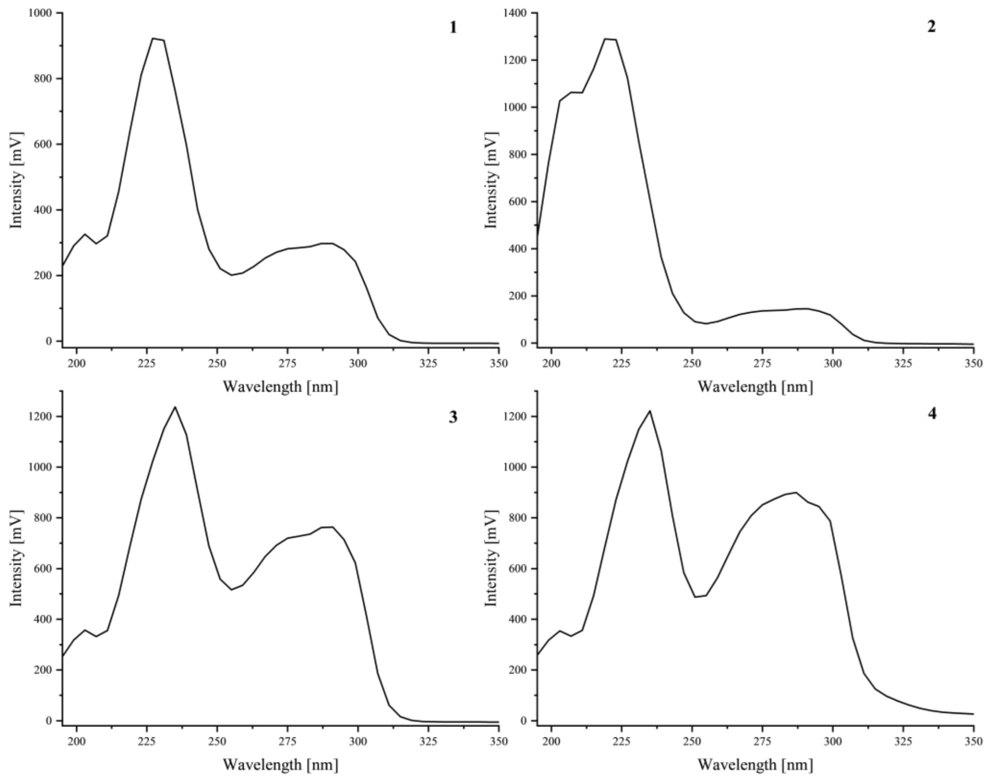

Figure S3 UV-spectra of all apicidins. Apicidin F (**1**) at λ_{max} = 230, 290 nm, apicidin J (**2**) with λ_{max} = 220, 291 nm, apicidin K (**3**) shows λ_{max} = 235, 291 nm, and apicidin L (**4**) with λ_{max} = 235, 287 nm. Spectra were determined during chromatographic separation with MeCN/ H_2O + 0.1% formic acid on a ReprosilPur C_{18}-AQ column (150 x 2 mm i.d., 3 µm particle size) equipped with a guard column of the same material (5 x 2 mm i.d.) using a linear gradient increasing from 10-100 % organic phase.

Apicidin F.

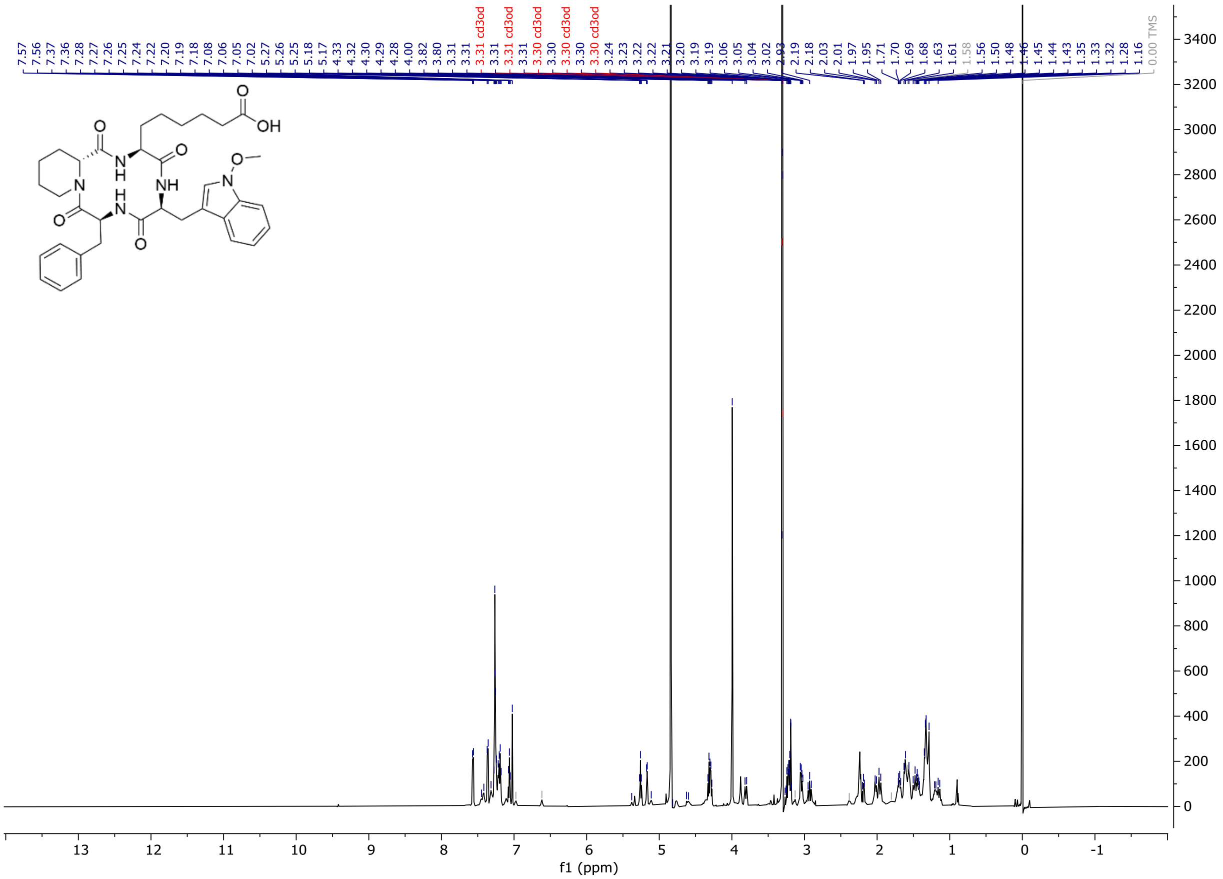

Figure S4 ^{1}H-NMR of apicidin F in CD_3OD at 600 MHz.

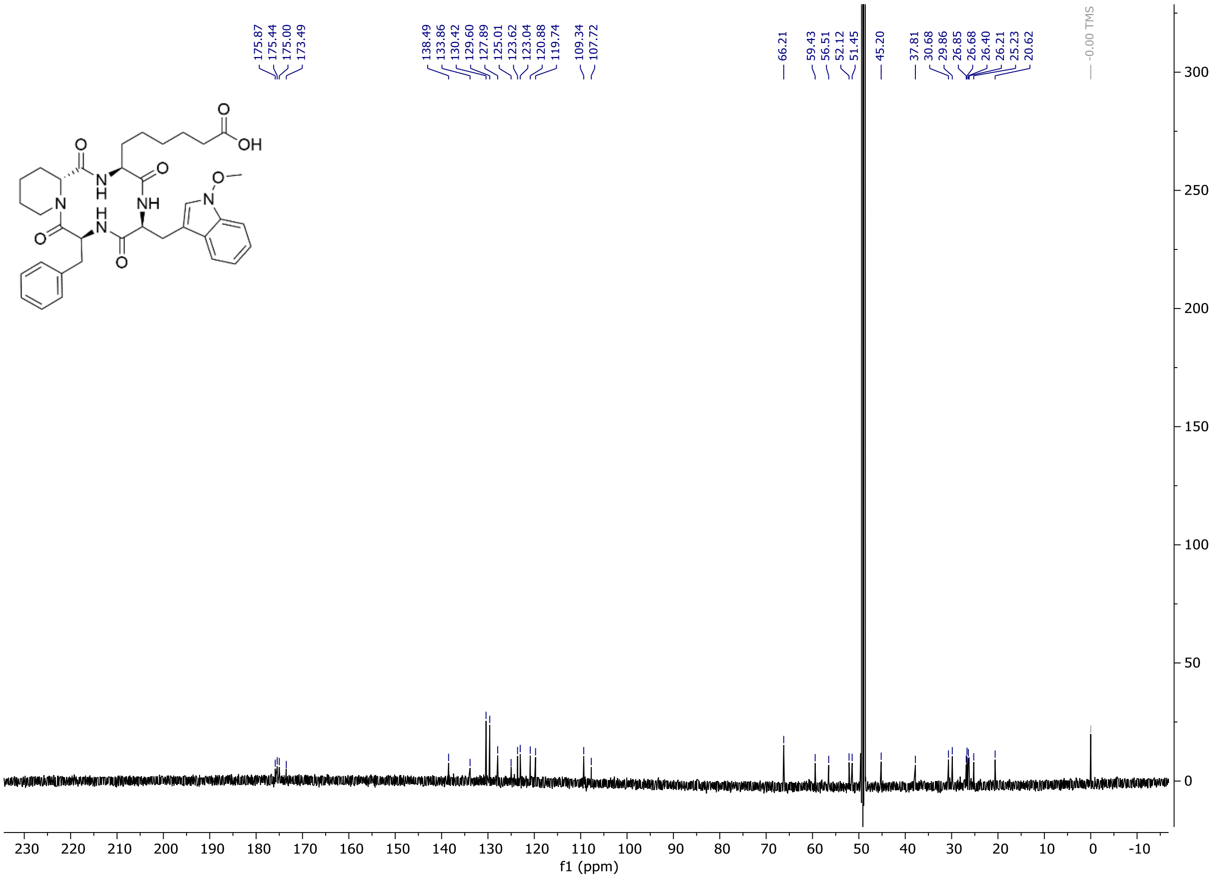

Figure S5 ^{13}C-NMR of apicidin F in CD_3OD at 150 MHz.

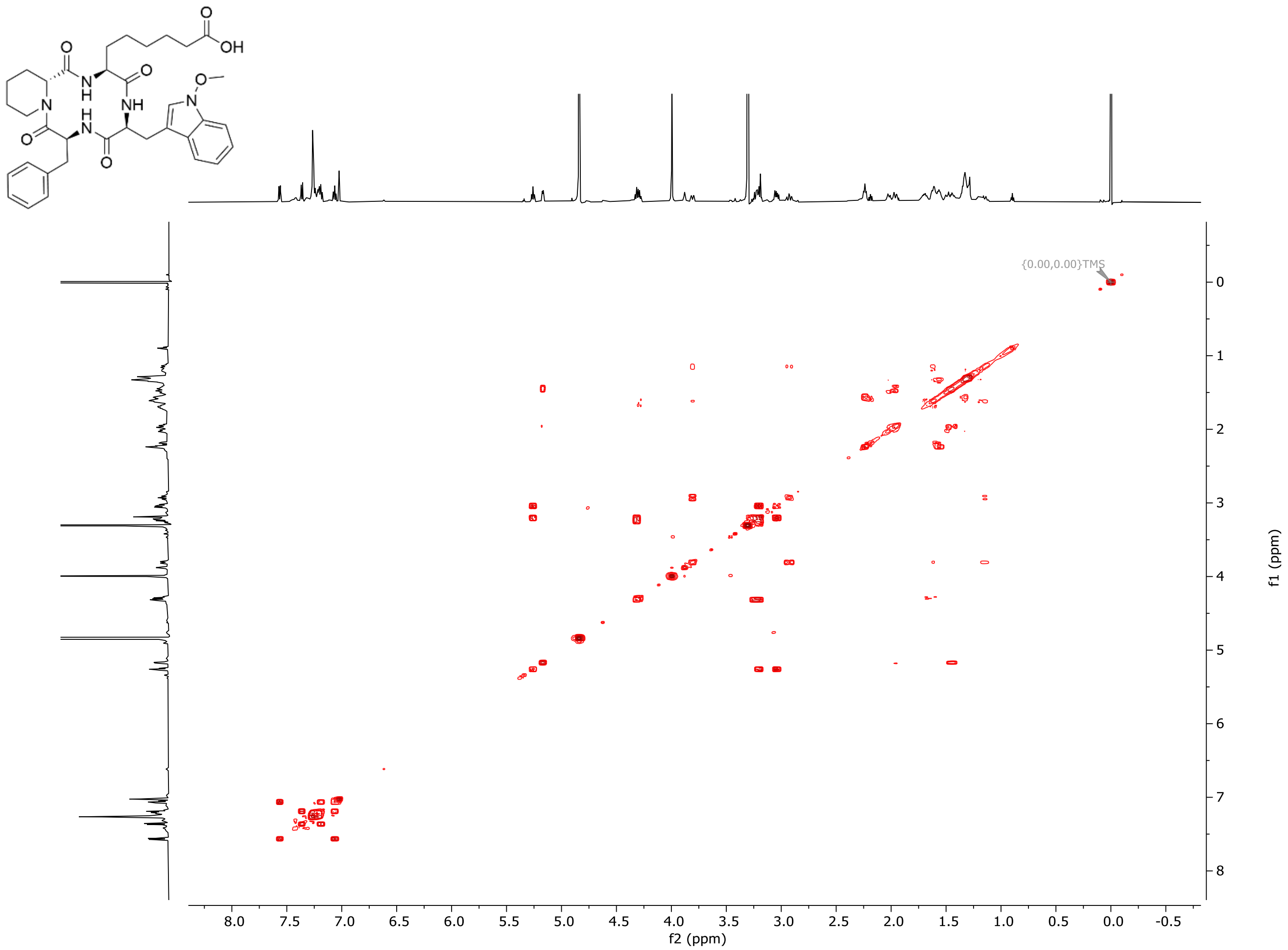

Figure S6 COESY-NMR of apicidin F in CD_3OD at 600 MHz.

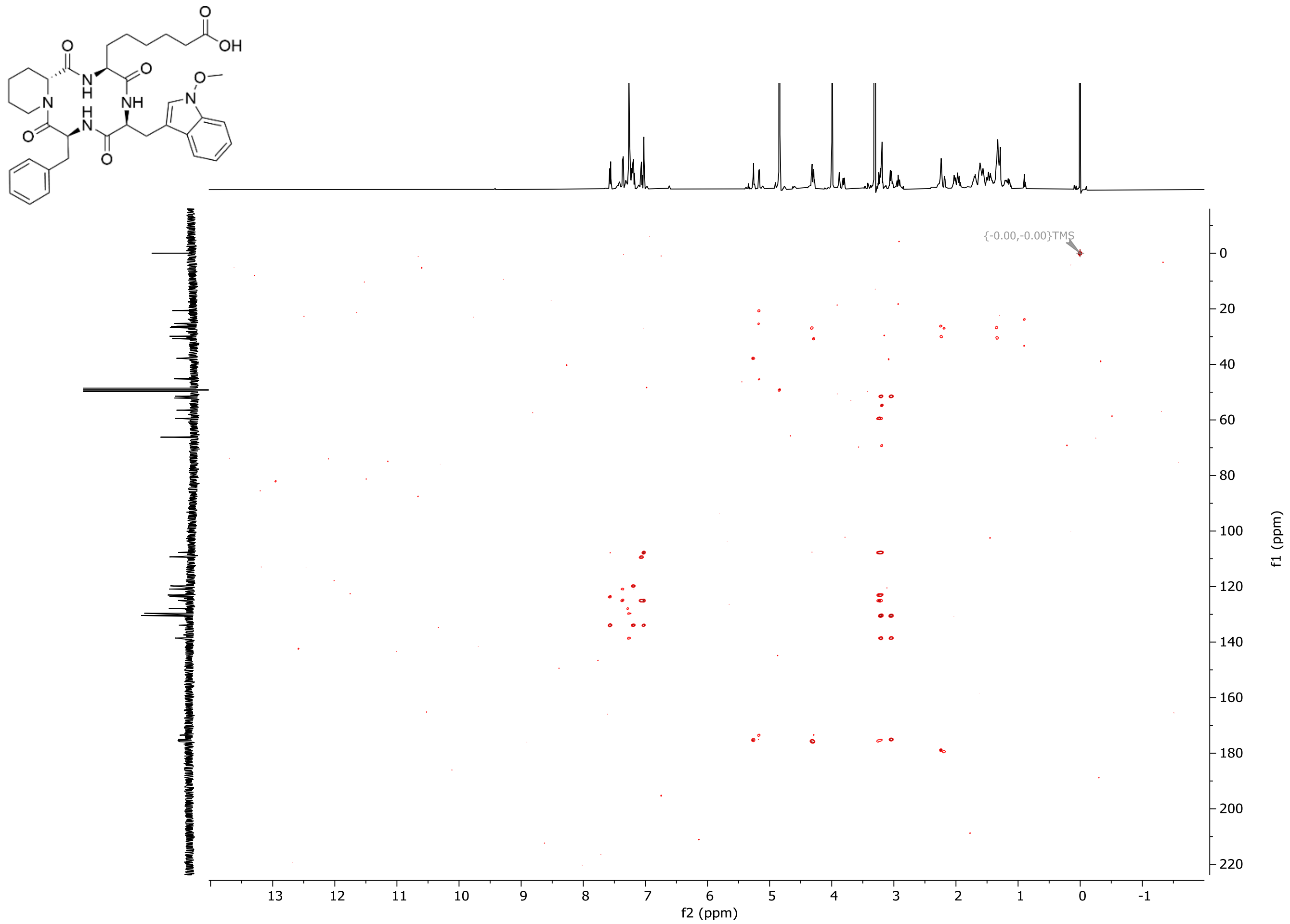

Figure S7 HMBC-NMR of apicidin F in CD_3OD at 150 and 600 MHz.

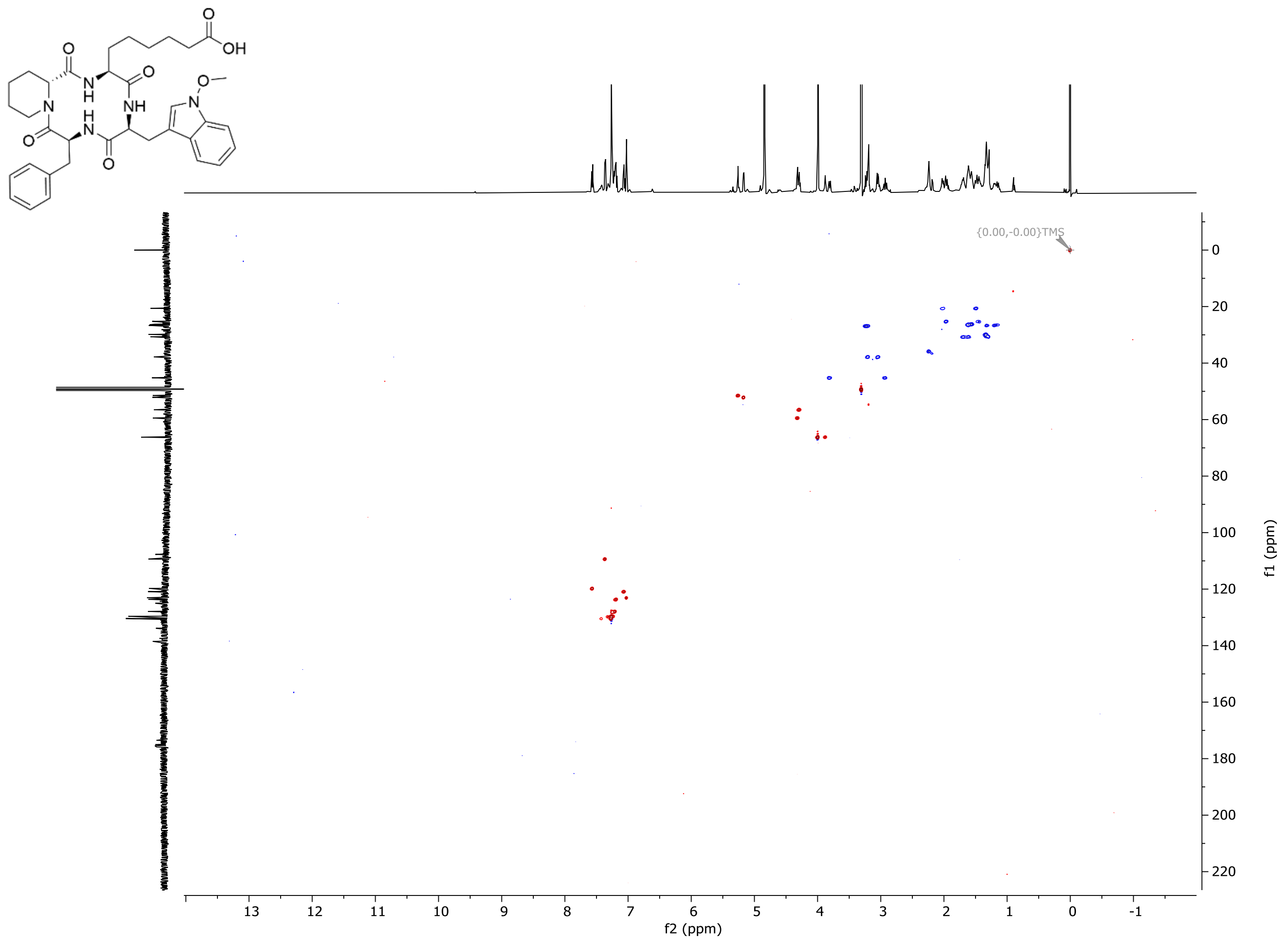

Figure S8 HSQC-NMR of apicidin F in CD_3OD at 150 and 600 MHz. Red signals show CH and CH_3 groups, blue signals are CH_2 signals.

Apicidin J.

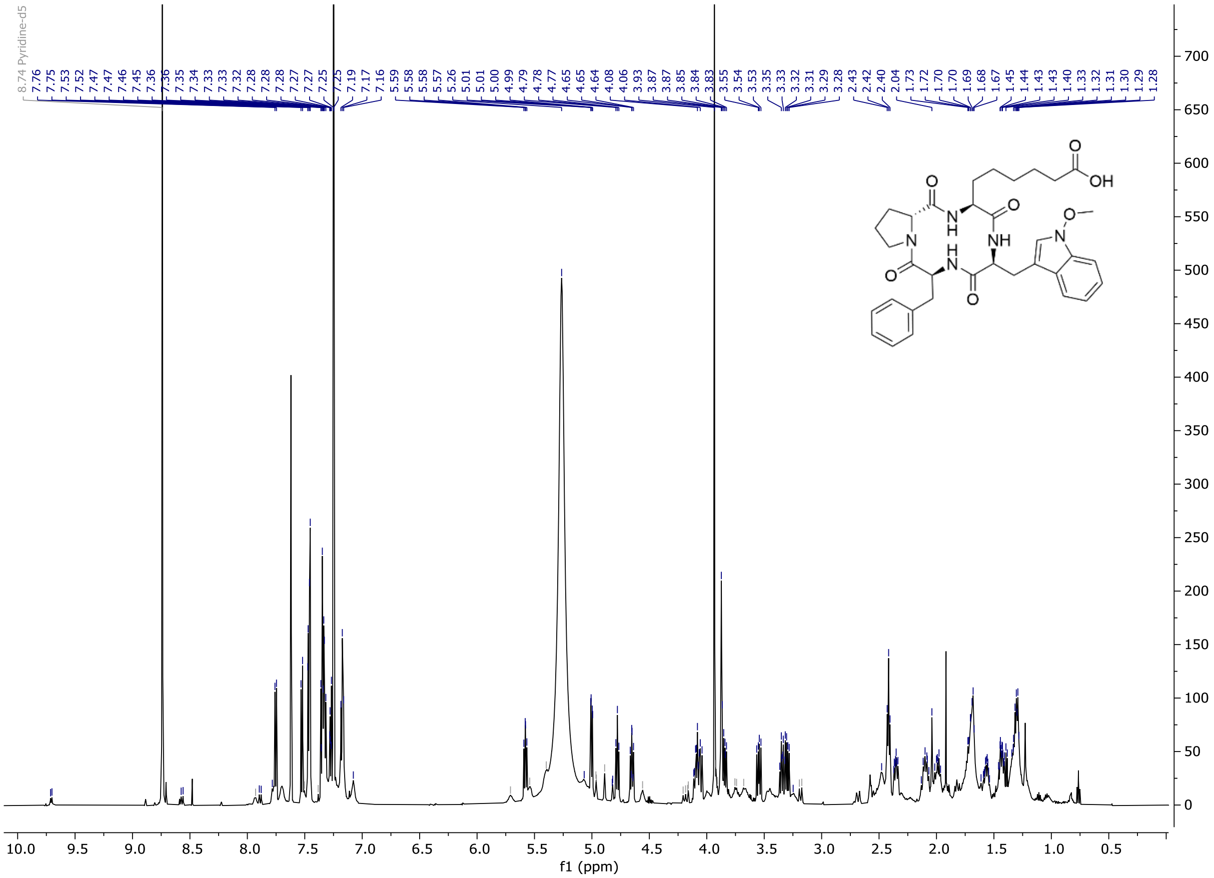

Figure S9 ^{1}H-NMR of apicidin J in C_5D_5N at 600 MHz.

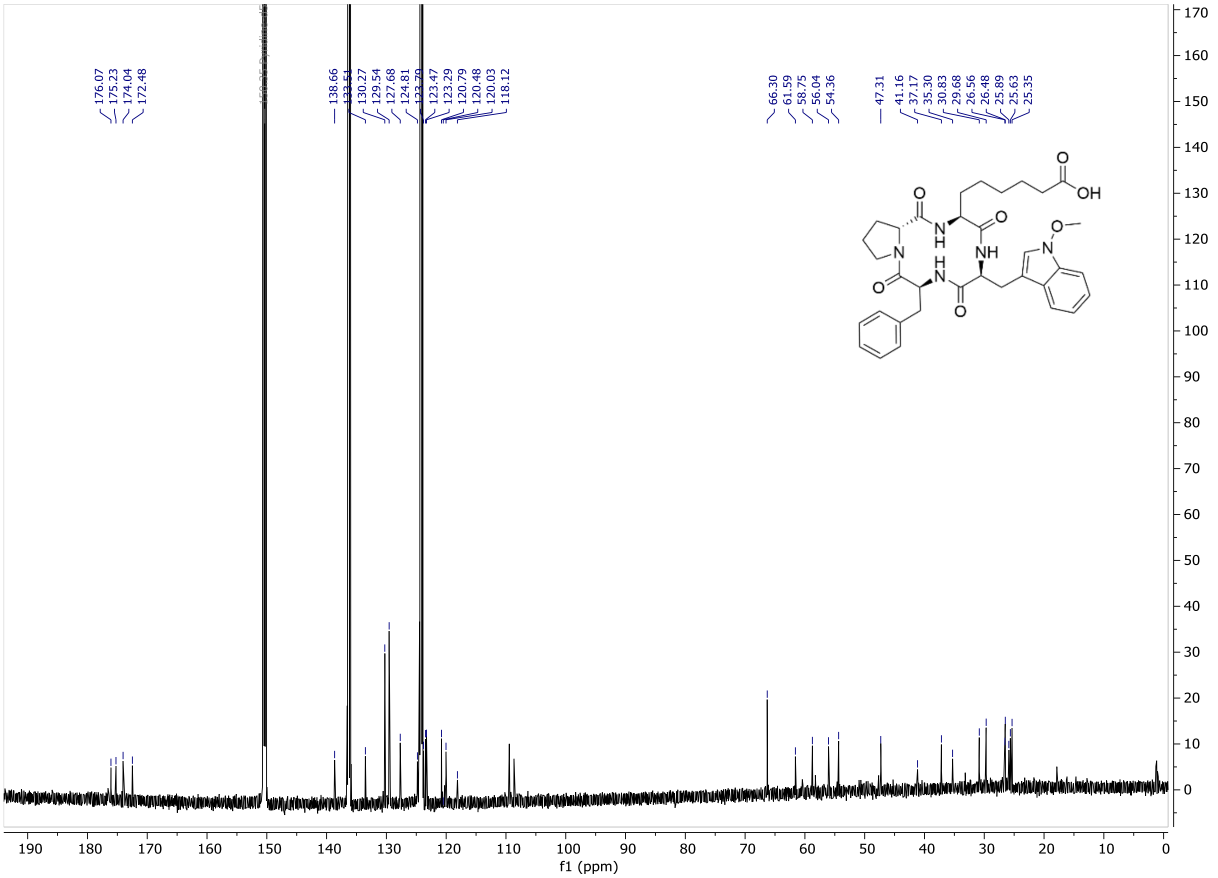

Figure S10 ^{13}C-NMR of apicidin J in C_5D_5N at 150 MHz.

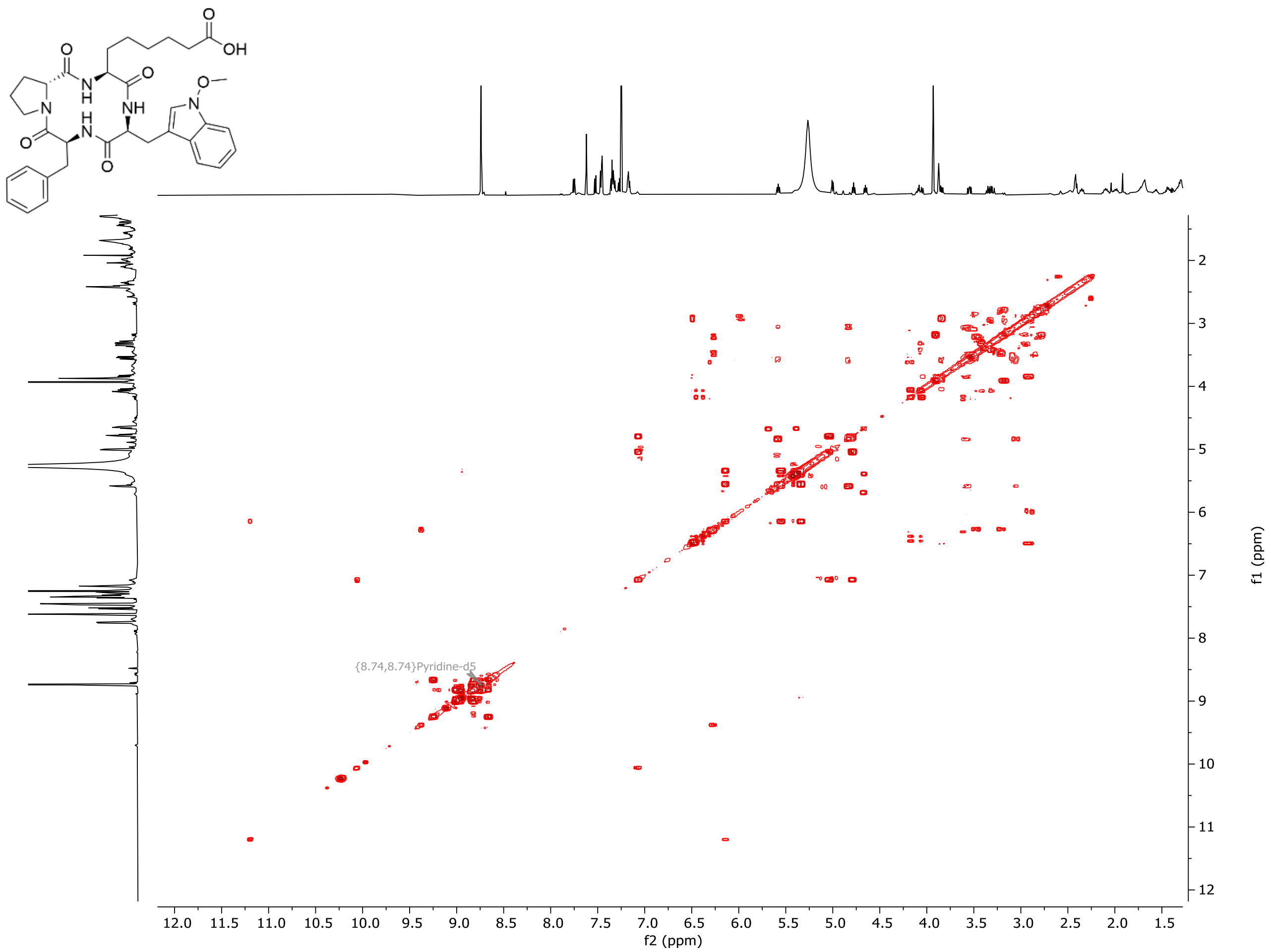

Figure S11 COESY-NMR of apicidin J in C_5D_5N at 600 MHz.

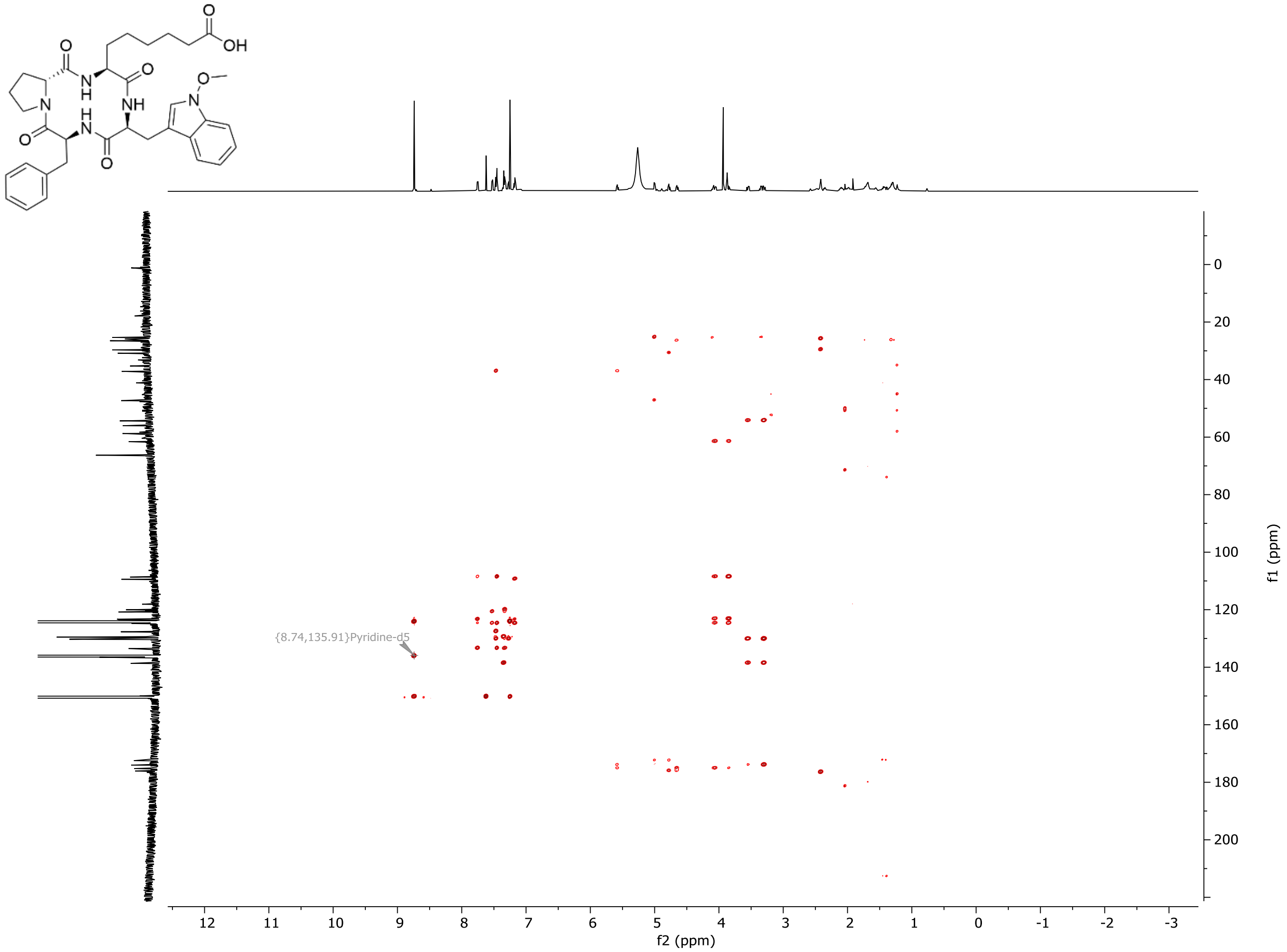

Figure S12 HMBC-NMR of apicidin J in C_5D_5N at 150 and 600 MHz.

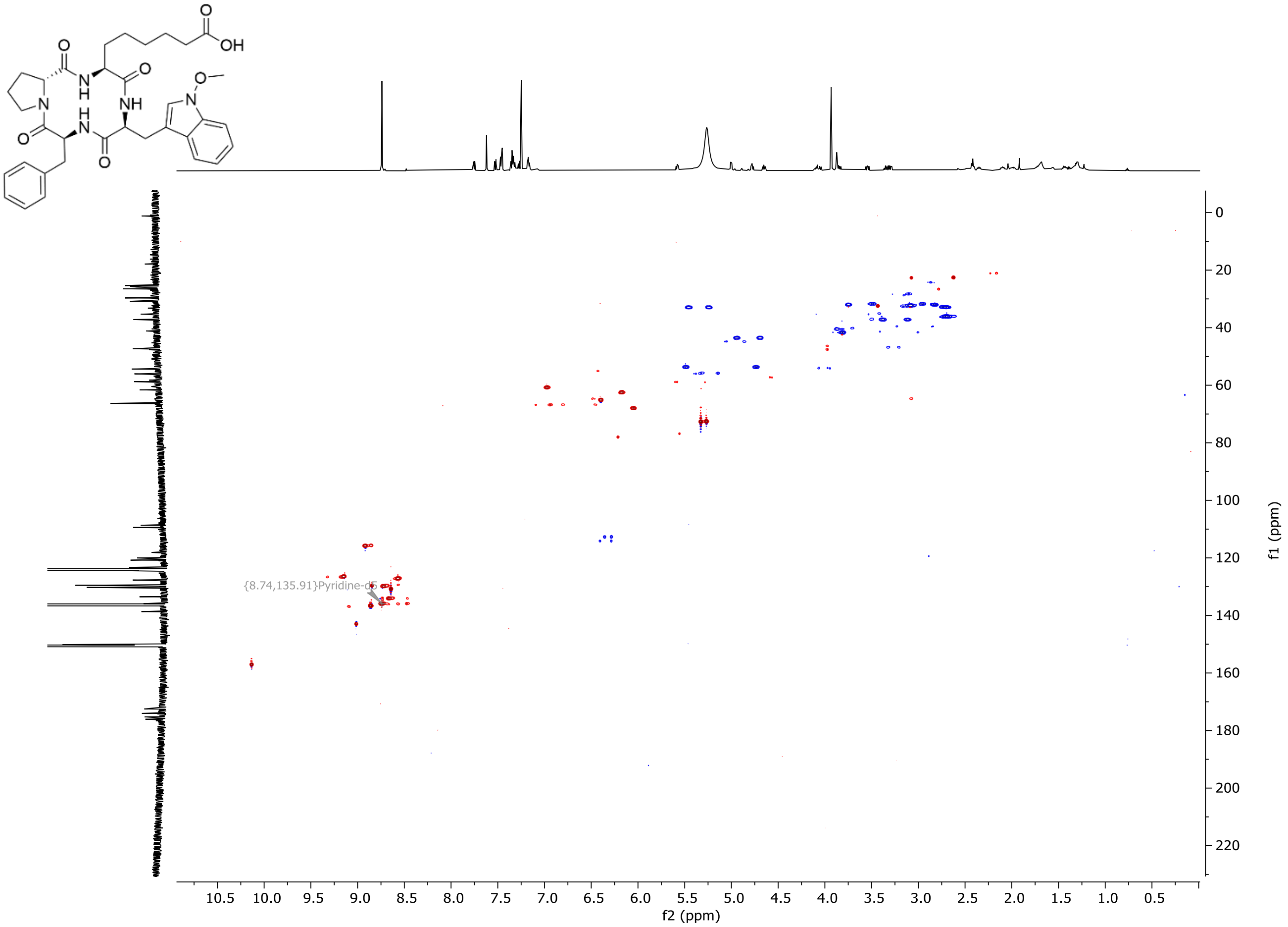

Figure S13 HSQC-NMR of apicidin J in C_5D_5N at 150 and 600 MHz. Red signals show CH and CH_3 groups, blue signals are CH_2 signals.

Apicidin K.

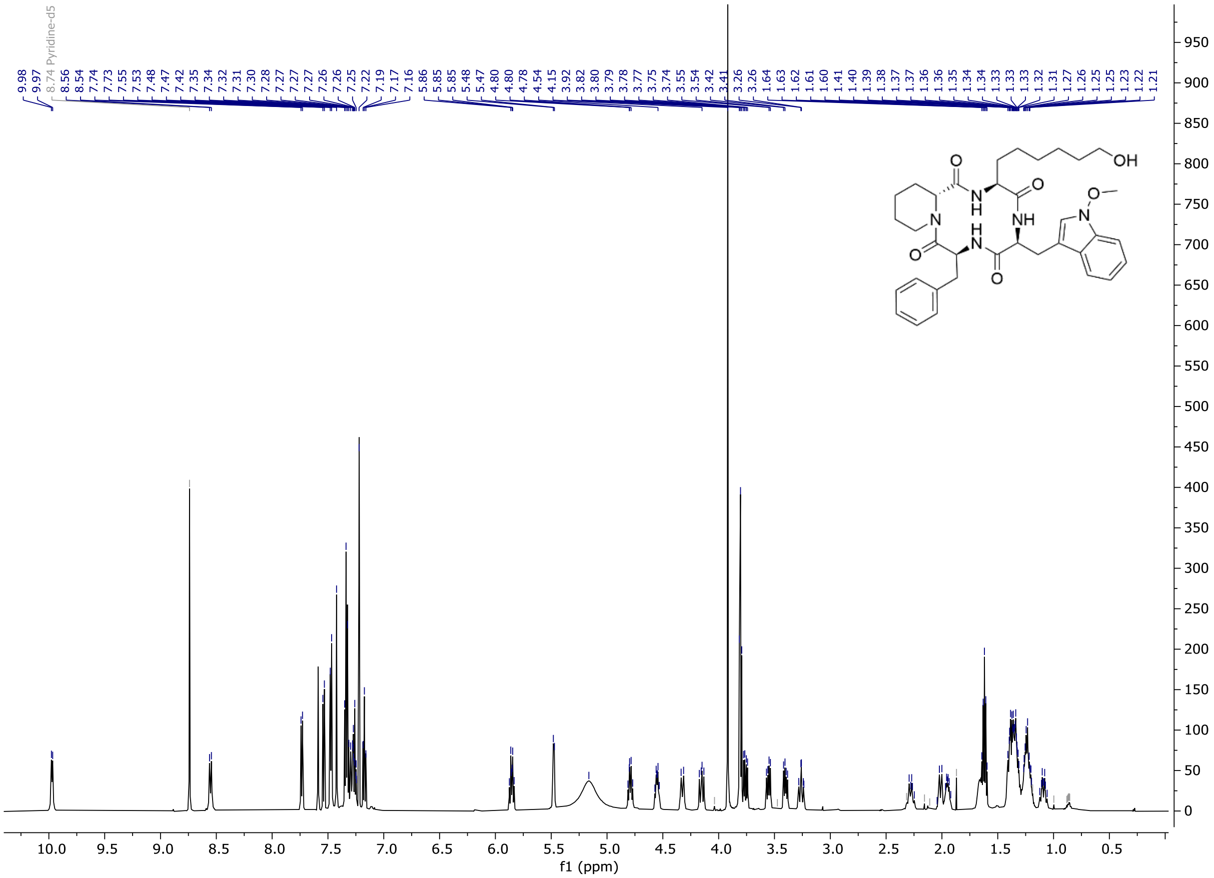

Figure S14 ^{1}H-NMR of apicidin K in C_5D_5N at 600 MHz.

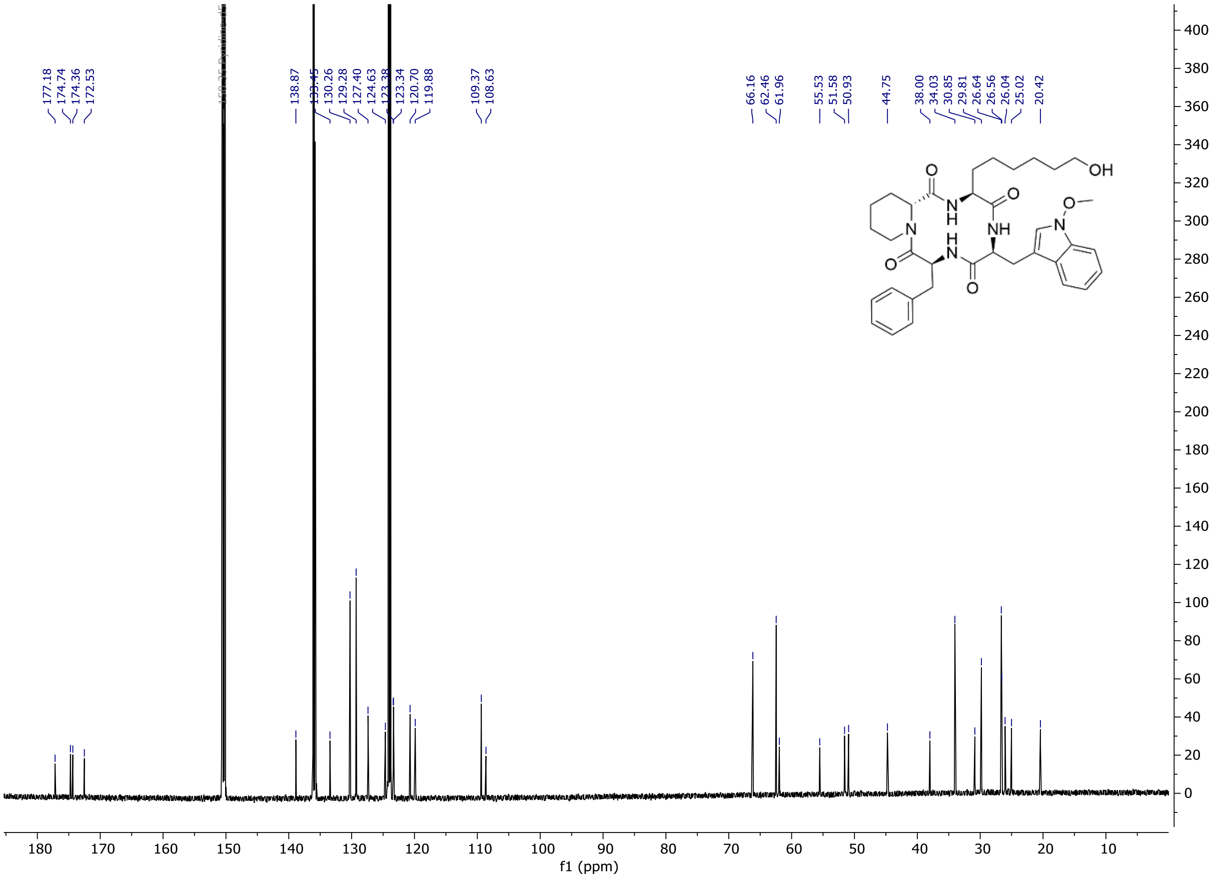

Figure S15 ^{13}C-NMR of apicidin K in C_5D_5N at 150 MHz.

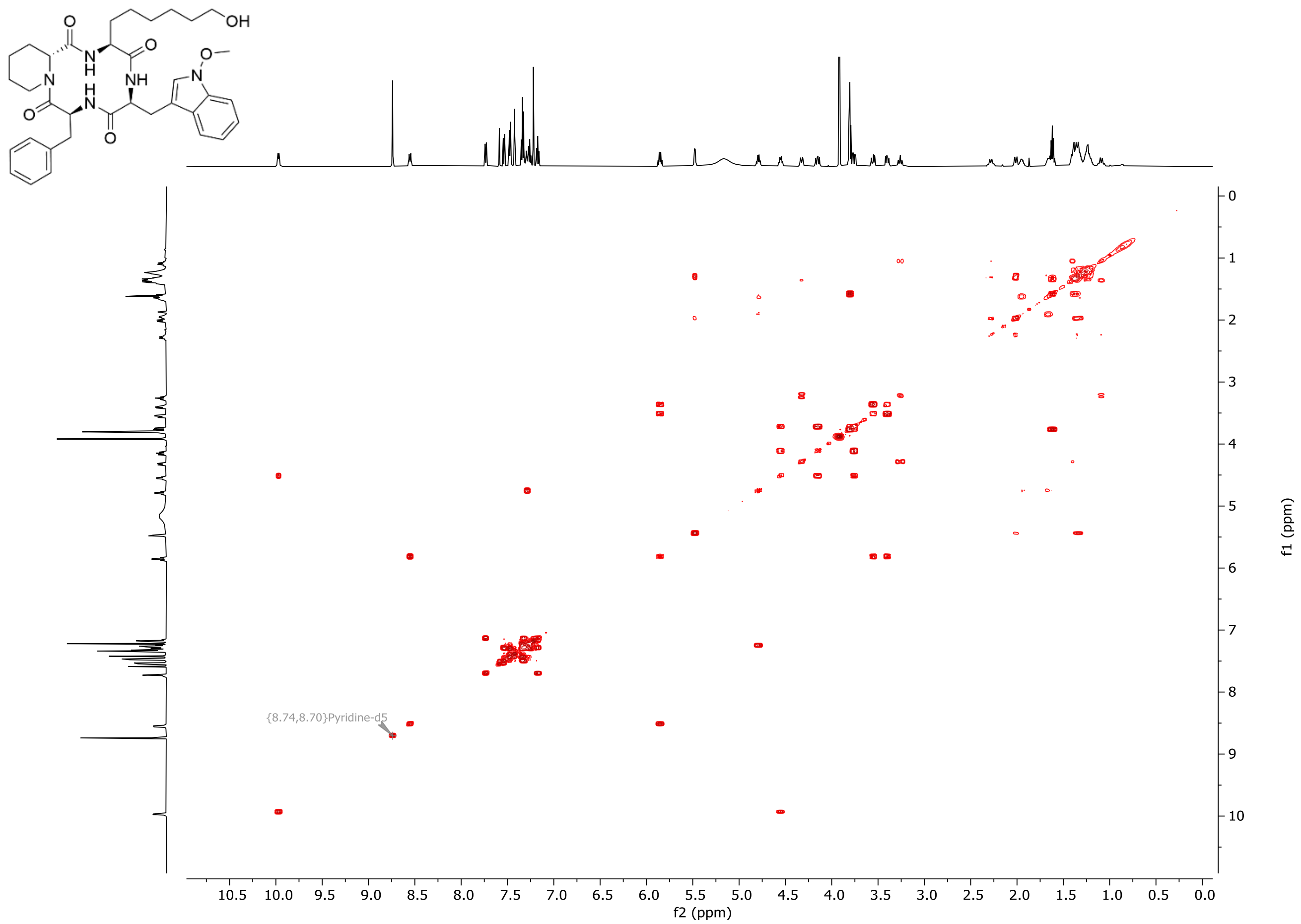

Figure S16 COESY-NMR of apicidin K in C_5D_5N at 600 MHz.

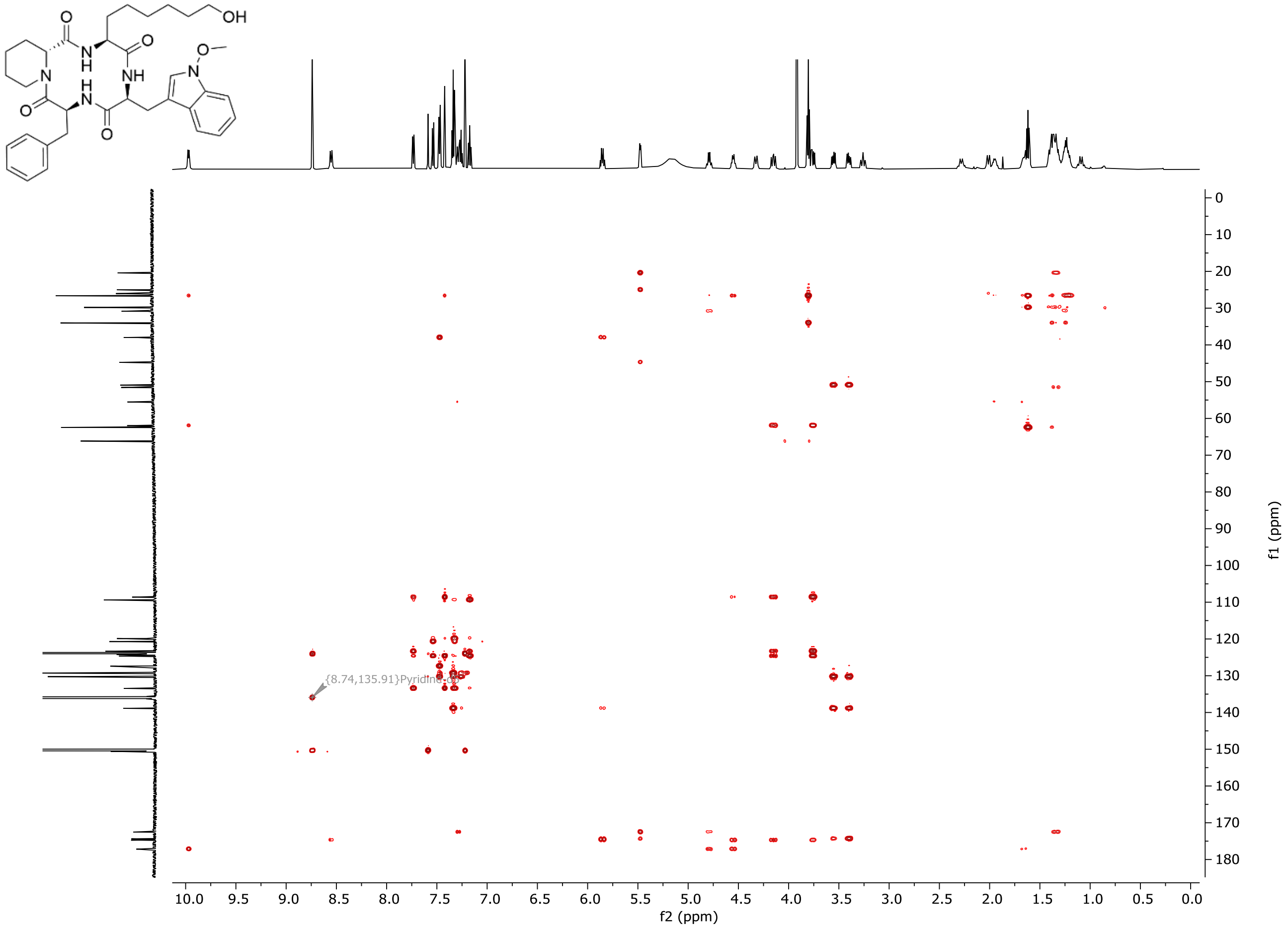

Figure S17 HMBC-NMR of apicidin K in C_5D_5N at 150 and 600 MHz.

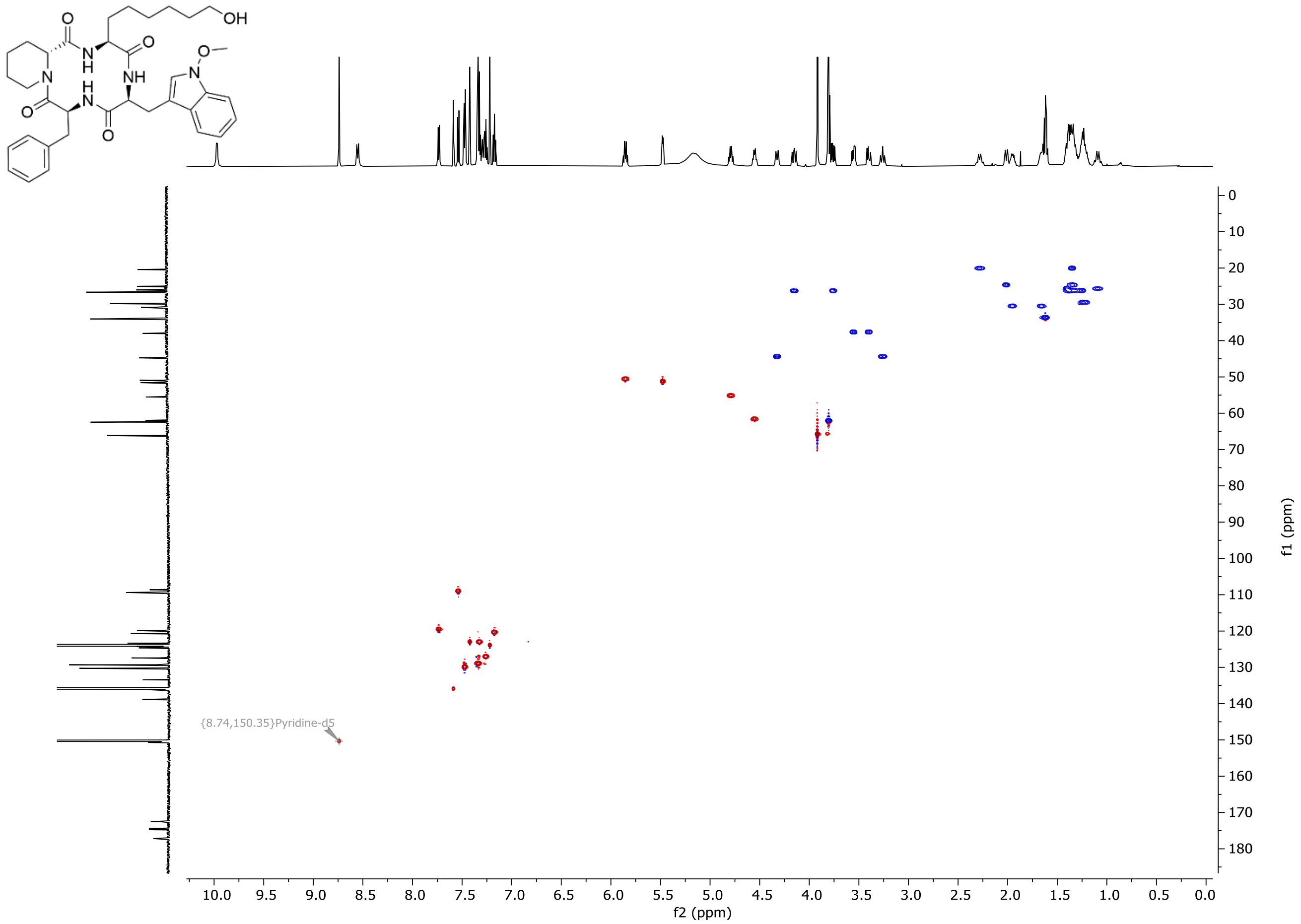

Figure S18 HSQC-NMR of apicidin K in C_5D_5N at 150 and 600 MHz. Red signals show CH and CH_3 groups, blue signals are CH_2 signals.

Apicidin L.

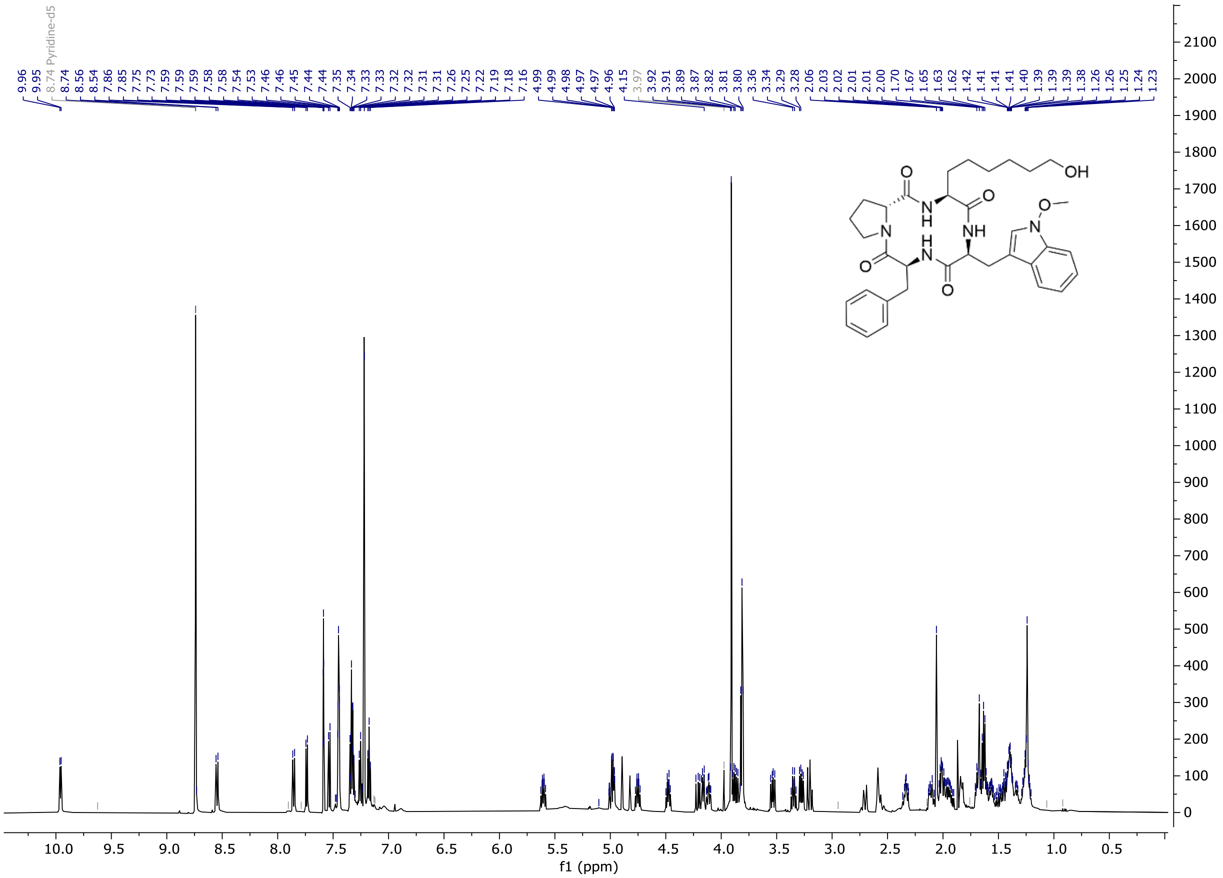

Figure S19 ^{1}H-NMR of apicidin L in C_5D_5N at 600 MHz.

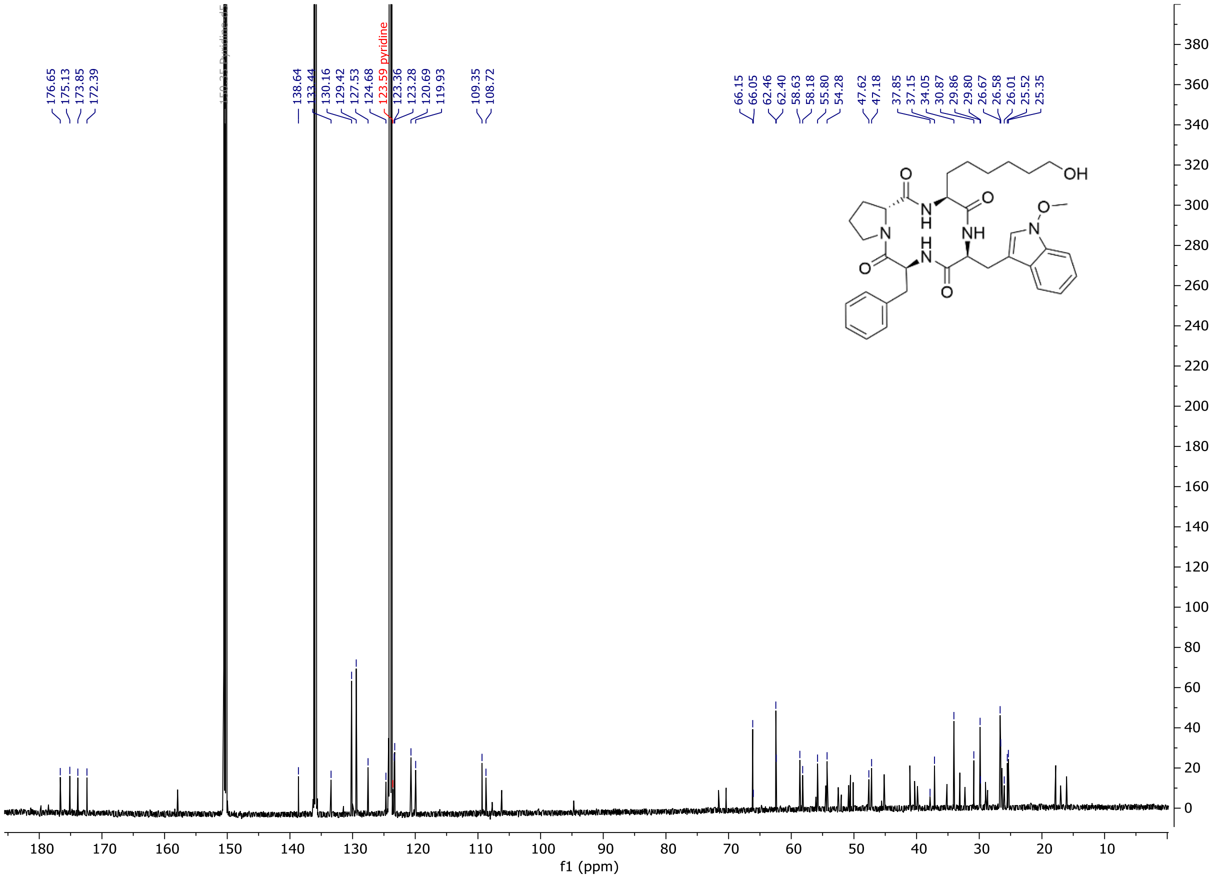

Figure S20 ^{13}C-NMR of apicidin L in C_5D_5N at 150 MHz.

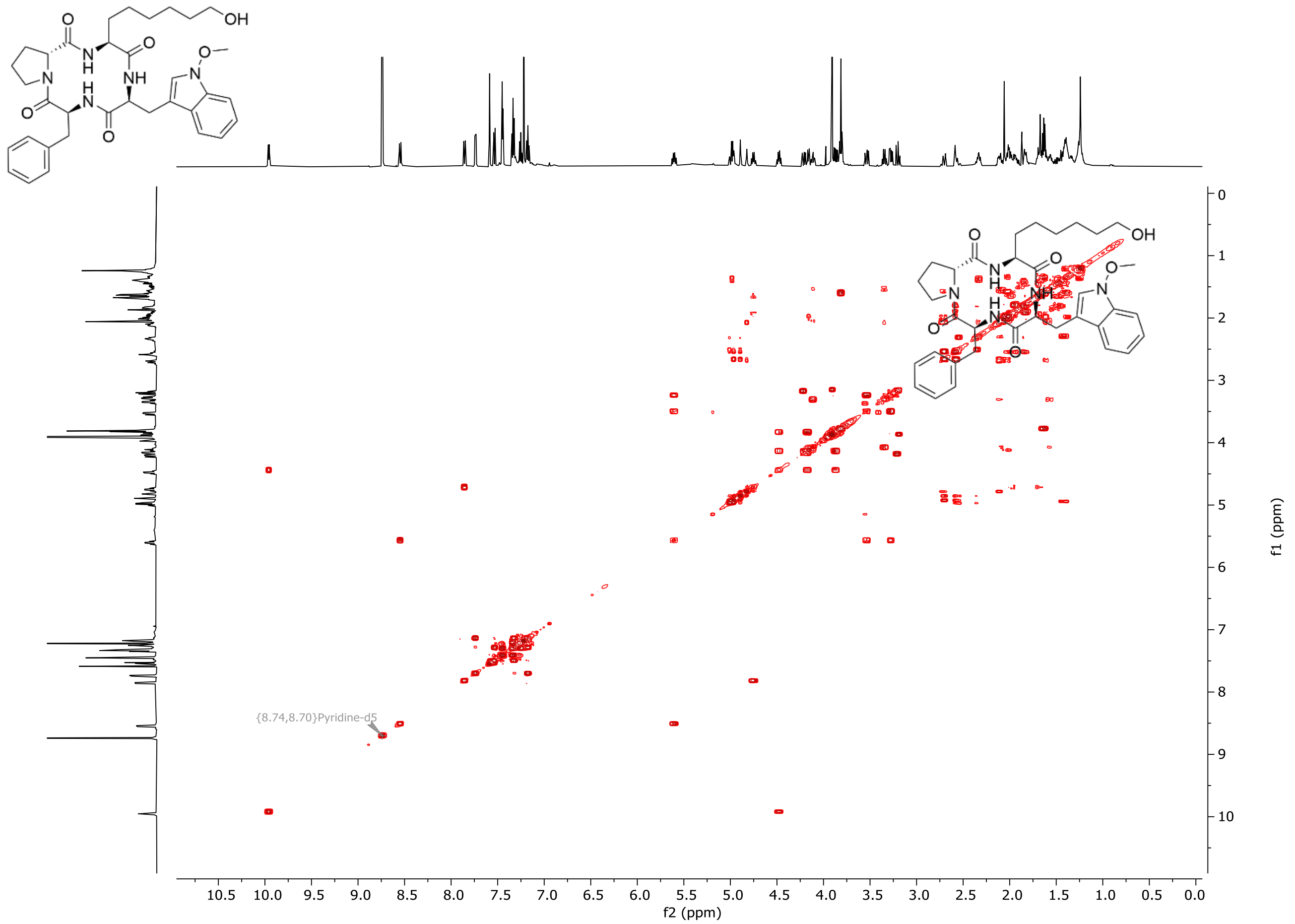

Figure S21 COESY-NMR of apicidin L in C_5D_5N at 600 MHz.

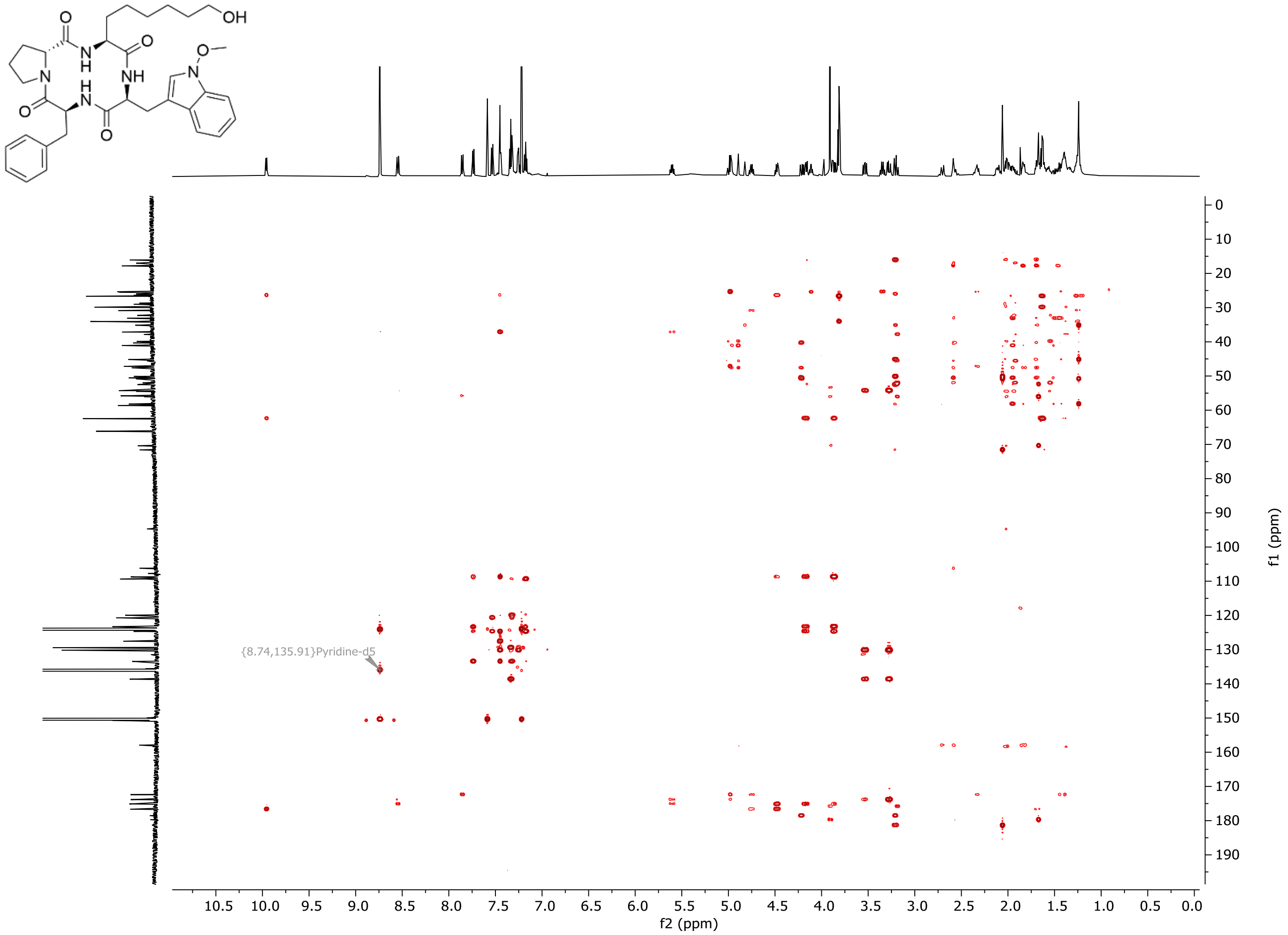

Figure S22 HMBC-NMR of apicidin L in C_5D_5N at 150 and 600 MHz.

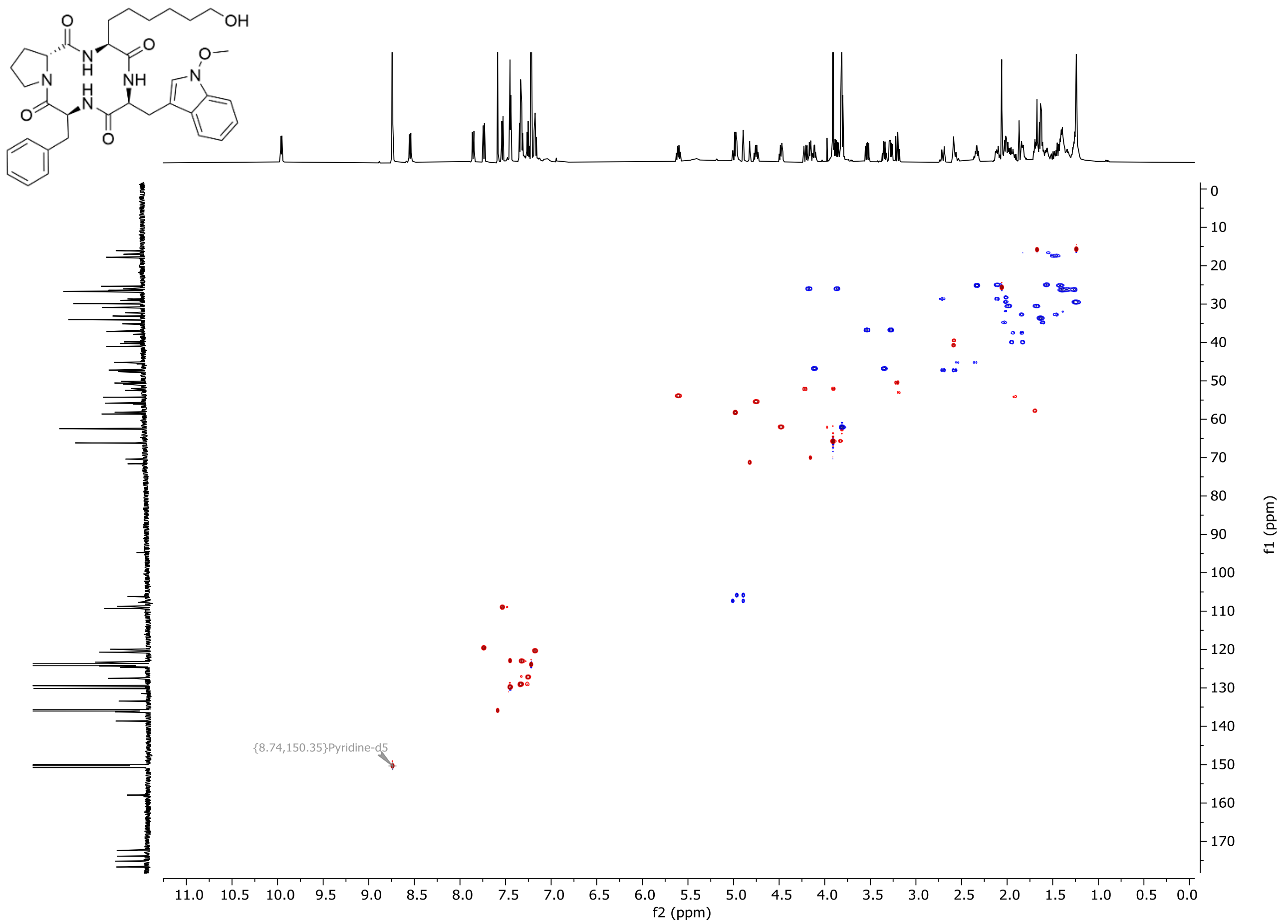

Figure S23 HSQC-NMR of apicidin L in C_5D_5N at 150 and 600 MHz. Red signals show CH and CH_3 groups, blue signals are CH_2 signals.

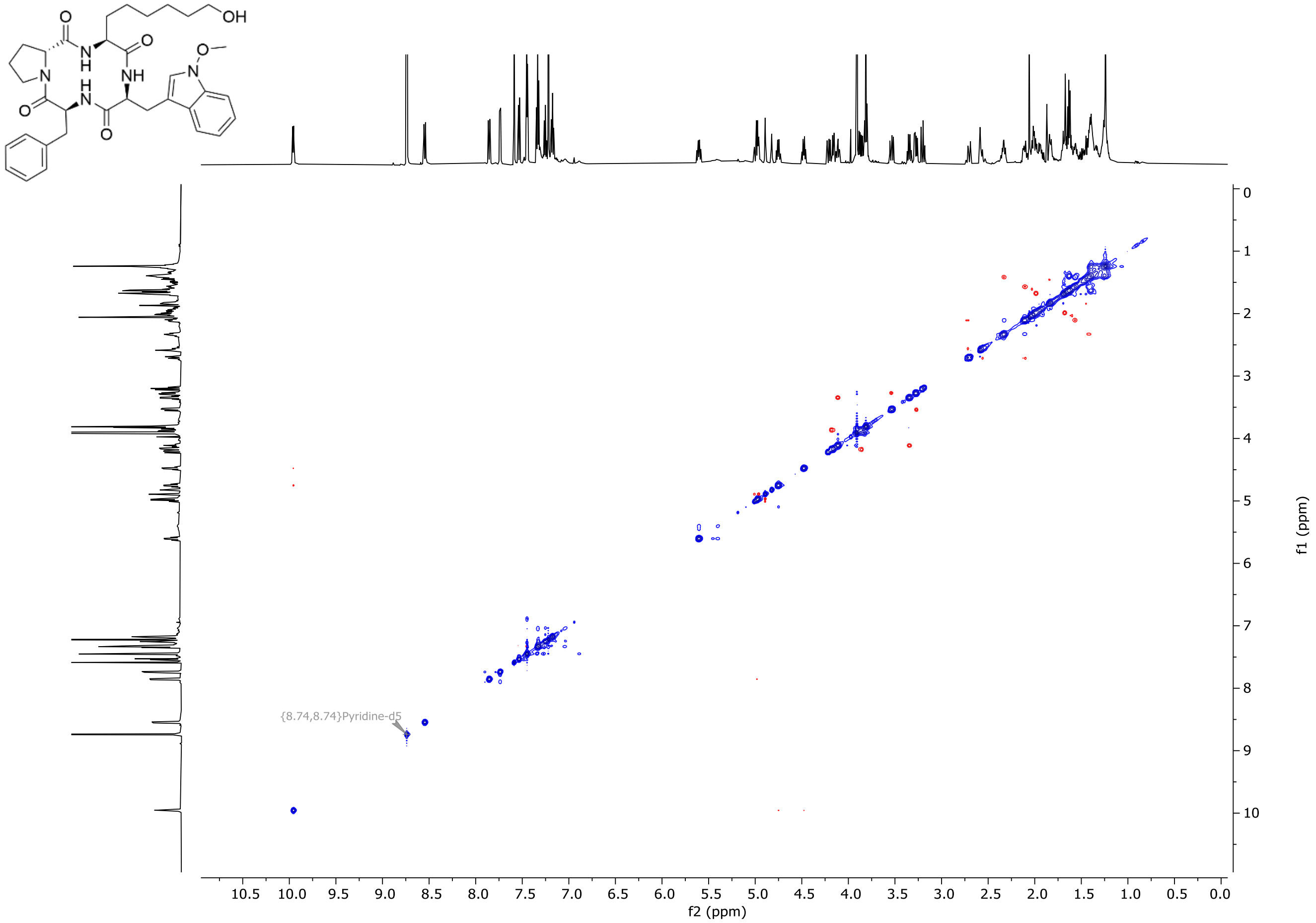

Figure S24 ROESY-NMR of apicidin L in C_5D_5N at 150 and 600 MHz. Red signals show ROESY correlations, blue signals are artifacts raised from COESY-correlations.

Equation S1 Calculation of mass error (Δm) in parts-per-million [ppm] (1) including an example calculation (2).

$$(1)\ \Delta m\ \ [ppm] = \frac{m/z_{\text{calculated}} - m/z_{\text{ measured}}}{m/z_{\text{ calculated}}} \times 10^6$$

$$(2)\ \Delta m\ \ [ppm] = \frac{618.3287 - 618.3278}{618.3287} \times 10^6 = 1.45\ ppm$$

Table S1 Overview of the MS^n fragmentation experiments. Precursor ion *m/z* is given with the mass error (Δm) in ppm together with the cum formula and the fragmentation energy given in % applied for collision-induced-dissociation (CID). All m/z are given as proton-adduct in positive electrospray-ionization mode ($[M+H]^+$), radical cationic fragments are implied as $[M+H]^{\cdot+}$. The sum formula of each proposed fragment structure is listed together with its respective calculated *m/z* and the Δm between measured and calculated *m/z* following Equation 1.

MS^n	Precursor ion *m/z* ± Δm, $[M+H]^+$, sum formula, CID energy	Measured fragment *m/z*, $[M+H]^+$	Sum formula	Calculated fragment *m/z* $[M+H]^+$	Δm [ppm]
MS^2	618.3278 ± 1.45 ppm<br>$C_{34}H_{44}N_5O_6^+$<br>11 %	590.3329	$C_{33}H_{44}N_5O_5^+$	590.3337	1.36
		588.3172	$C_{33}H_{42}N_5O_5^+$	588.3181	1.53
		587.3093	$C_{33}H_{41}N_5O_5^{\cdot+}$	587.3103	1.70
		586.3015	$C_{33}H_{40}N_5O_5^+$	586.3024	1.54
		558.3066	$C_{32}H_{40}N_5O_4^+$	558.3075	1.61
		458.2518	$C_{24}H_{34}N_4O_5^{\cdot+}$	458.2524	1.31
		332.1390	$C_{20}H_{18}N_3O_2^+$	332.1394	1.20
MS^3	586.3014 ± 1.71 ppm<br>$C_{33}H_{40}N_5O_6^+$<br>13 %	558.3065	$C_{32}H_{40}N_5O_4^+$	558.3075	1.70
		429.1915	$C_{25}H_{25}N_4O_3^+$	429.1922	1.63
		332.1389	$C_{20}H_{18}N_3O_2^+$	332.1394	1.51
		304.1441	$C_{19}H_{18}N_3O^+$	304.1445	1.32
MS^4	558.3064 ± 1.79 ppm<br>$C_{32}H_{40}N_5O_4^+$<br>10 %	444.2275	$C_{27}H_{30}N_3O_3^+$	444.2282	1.58
		429.1914	$C_{25}H_{25}N_4O_3^+$	429.1922	1.86
		332.1389	$C_{20}H_{18}N_3O_2^+$	332.1394	1.51
MS^4	332.1387 ± 0.90 ppm<br>$C_{20}H_{18}N_3O_2^+$<br>12 %	304.1436	$C_{19}H_{18}N_3O^+$	304.1445	1.32

HRMS fragmentation spectra. Apicidin L at 15 µg/mL in MeCN/H_2O (80/20, v/v) + 1 % acetic acid, observed $[M+H]^+$ *m/z* 618.3278.

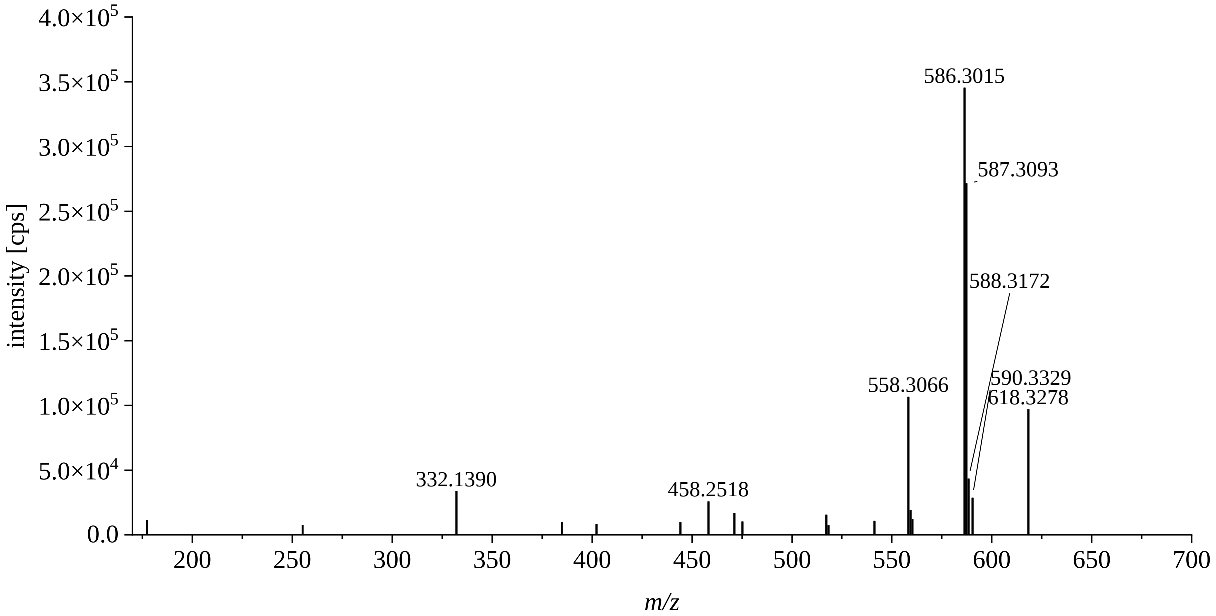

Figure S25 MS^2 spectrum of apicidin L in positive HESI from *m/z* 100-700. Parent ion is *m/z* 618.3278, CID at 11 %.

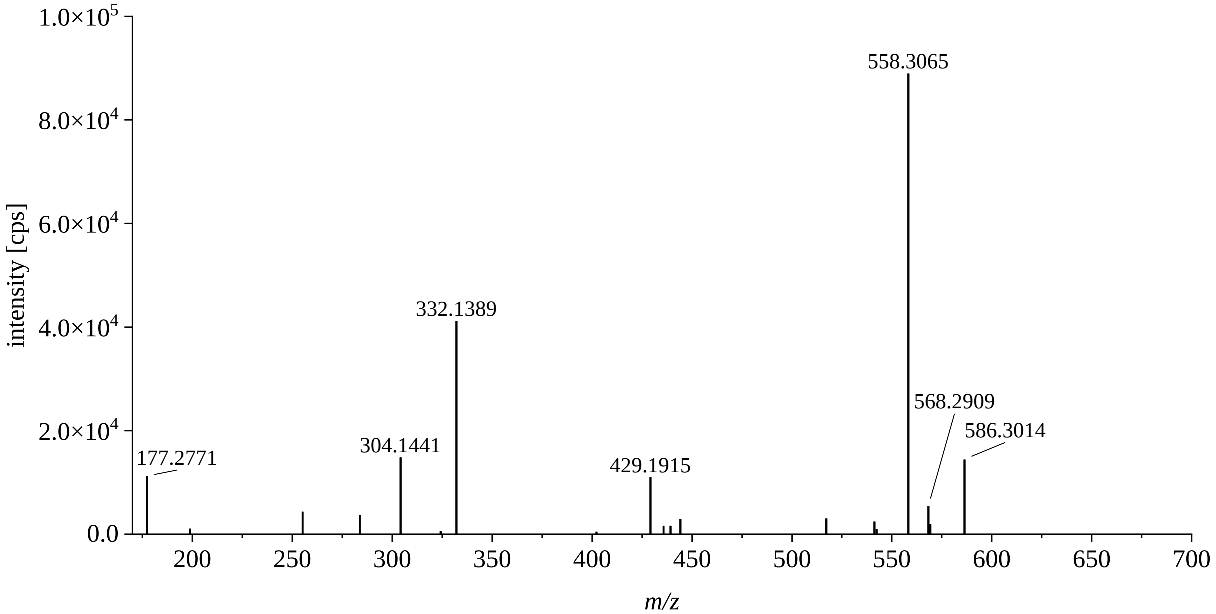

Figure S26 MS^3 spectrum of apicidin L in positive HESI from *m/z* 100-700. Parent ion is *m/z* 586.3065, CID at 13 %.

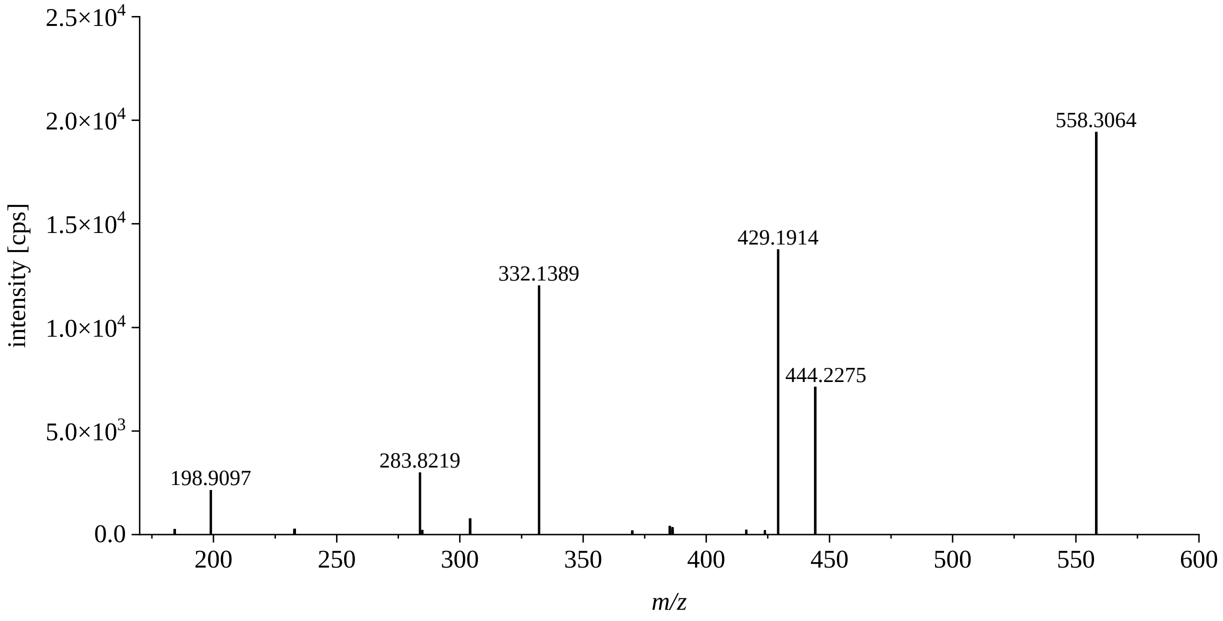

Figure S27 MS^4 spectrum of apicidin L in positive HESI from *m/z* 100-600. Parent ion *m/z* is 558.3064, CID at 12 %.

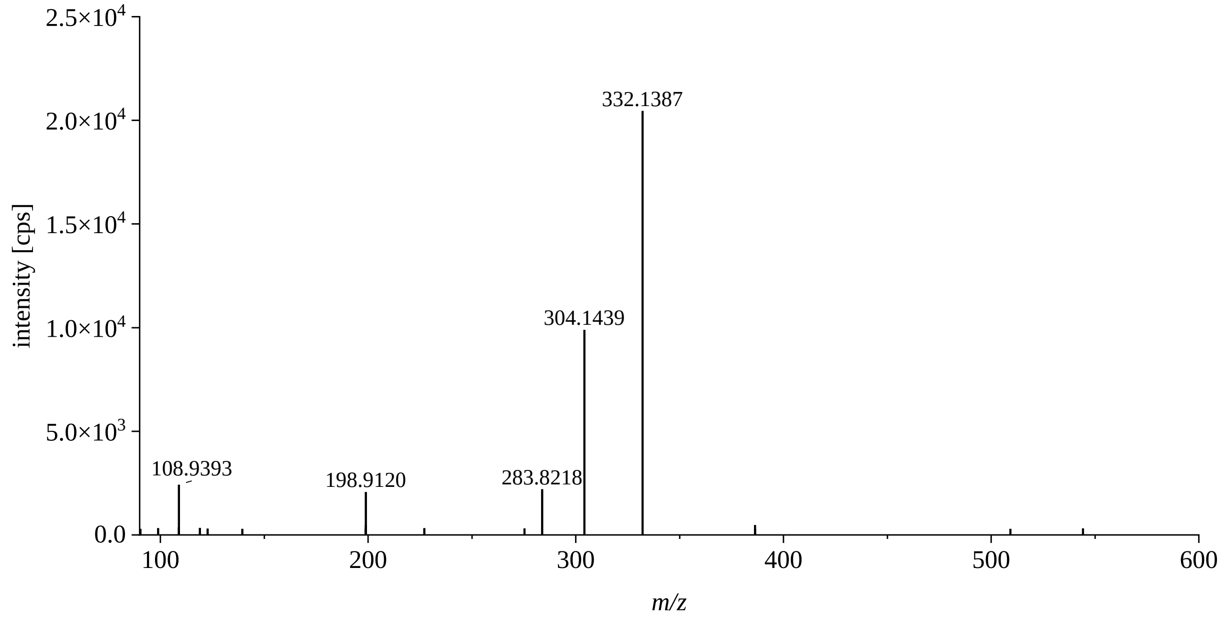

Figure S28 MS^4 spectrum of apicidin L in positive HESI from *m/z* 100-600. Parent ion is *m/z* 332.1387, CID at 10 %.
